# Transcriptional pathways of terminal differentiation in high- and low-density blood granulocytes in sepsis

**DOI:** 10.1186/s12950-024-00414-w

**Published:** 2024-10-21

**Authors:** Tobias Guenther, Anna Coulibaly, Sonia Y. Velásquez, Jutta Schulte, Tanja Fuderer, Timo Sturm, Bianka Hahn, Manfred Thiel, Holger A. Lindner

**Affiliations:** Department of Anesthesiology, Surgical Intensive Care Medicine and Pain Medicine, Mannheim Institute of Innate Immunoscience (MI3), Medical Faculty Mannheim, Heidelberg University, Mannheim, Germany

**Keywords:** Biomarkers, Gene expression, Granulocyte precursor cells, Sepsis, Systemic inflammatory response syndrome, SeptiCyte™ lab

## Abstract

**Background:**

Trauma and infection induce emergency granulopoiesis. Counts of immature granulocytes and transcriptional pathways of terminal granulocytic differentiation in blood are elevated in sepsis but correlate with disease severity. This limits their performance as sepsis biomarkers in critically ill patients. We hypothesized that activation of these pathways in sepsis is attributable to immature low-density (LD) rather than mature high-density (HD) granulocytes.

**Methods:**

We included patients with sepsis and systemic inflammatory response syndrome (SIRS) of comparable disease severity, and additionally septic shock, on intensive or intermediate care unit admission. Blood granulocyte isolation by CD15 MicroBeads was followed by density-gradient centrifugation. Flow cytometry was used to determine counts of developmental stages (precursors) and their relative abundancies in total, HD, and LD granulocytes. Five degranulation markers were quantified in plasma by multiplex immunoassays. A set of 135 genes mapping granulocyte differentiation was assayed by QuantiGene™ Plex. *CEACAM4*, *PLAC8*, and *CD63* were analyzed by qRT-PCR. Nonparametric statistical tests were applied.

**Results:**

Precursor counts appeared higher in sepsis than SIRS but did not correlate with disease severity for early immature and mature granulocytes. Precursor subpopulations were enriched at least ten-fold in LD over HD granulocytes without sepsis-SIRS differences. Degranulation markers in blood were comparable in sepsis and SIRS. Higher expression of early developmental genes in sepsis than SIRS was more pronounced in LD and less in HD than total granulocytes. Only the cell membrane protein encoding genes *CXCR2* and *CEACAM4* were more highly expressed in SIRS than sepsis. By qRT-PCR, the azurophilic granule genes *CD63* and *PLAC8* showed higher sepsis than SIRS levels in LD granulocytes and *PLAC8* also in total granulocytes where its discriminatory performance resembled C-reactive protein (CRP).

**Conclusions:**

Transcriptional programs of early terminal granulocytic differentiation distinguish sepsis from SIRS due to both higher counts of immature granulocytes and elevated expression of early developmental genes in sepsis. The sustained expression of *PLAC8* in mature granulocytes likely accounts for its selection in the whole blood SeptiCyte™ LAB test. Total granulocyte *PLAC8* rivals CRP as sepsis biomarker. However, infection-specific transcriptional pathways, that differentiate sepsis from sterile stress-induced granulocytosis more reliably than CRP, remain to be identified.

**Supplementary Information:**

The online version contains supplementary material available at 10.1186/s12950-024-00414-w.

## Background

Sepsis is caused by a dysregulated host response to infection and is a leading cause of global morbidity and mortality [1]. In bacterial sepsis, timely source control and administration of antibiotics are life-saving [2]. In the absence of a rapid gold-standard diagnostic test, the early distinction of infectious from non-infectious life-threatening deterioration is challenging. This is especially true in the critically ill, where systemic inflammatory response syndrome (SIRS) and organ dysfunction, i.e., clinical criteria for infection and sepsis, are both highly prevalent [3]. Confirmation of infection by microbiology testing takes hours to days [4]. To date, the diagnosis of sepsis thus largely relies on clinical gestalt [5]. In sepsis, routine clinical data are prognostic of patient outcomes, but their use for early recognition of infection and sepsis is limited [6].

Several multigene expression classifiers for infection and sepsis, identified from whole blood transcriptomics, promise to support early diagnosis and outcome prediction and to unveil underlying biological processes [7–10]. One of them, the SeptiCyte™ LAB test [11, 12], has obtained U.S. Food and Drug Administration clearance [13]. Importantly, whole blood gene signatures of sepsis commonly correlate with disease severity and mortality, which can also result from a dysregulated host response to sterile tissue damage [6, 14]. It is unclear, to what degree known sepsis gene signatures are attributable to the original infection or ensuing tissue damage. Particularly, reported whole blood gene signatures of acute respiratory distress syndrome [15–17] and non-resolving multiple organ dysfunction [18] commonly feature several marker genes of early terminal granulocytic differentiation indicative of emergency granulopoiesis [19]. Emergency granulopoiesis in response to severe infection and trauma contributes to second-organ damage and thus aggravates disease severity in either condition [20, 21]. In sepsis, it may also further increase infection risk compared to healthy controls due to immunosuppressive properties of neutrophils [22].

Instead of whole blood, we recently compared the transcriptomes of isolated CD15^+^ blood leukocytes of patients with SIRS and sepsis on admission to an interdisciplinary surgical intensive care unit (ICU) [19]. These cells encompass the stages of normal granulocyte maturation in the bone marrow beyond the myeloblast stage [23]. Higher blood counts as well as higher expression of promyelocyte- and myelocyte-restricted genes and pathways of these early granulocyte precursors indeed distinguished sepsis from SIRS [19]. However, sequential organ failure assessment (SOFA) scores were significantly higher in patients with sepsis than with SIRS so tissue damage may also have contributed to the blood count and transcriptional differences in these patients.

In this study, we examined reported gene signatures of early terminal granulocytic differentiation in sepsis and SIRS [19] using transcriptional profiling of total and density gradient separated granulocytes. By this preparation method, mature and immature granulocytes are enriched in the high- and low-density (HD and LD) fraction, respectively [24]. We enrolled patients with sepsis and SIRS of comparable disease severity on ICU and intermediate care unit (IMC) admission. The granulocytic series was characterized by flow cytometry and granulocyte degranulation markers by multiplex immunoassays. Key signature genes and pathways of terminal granulocytic differentiation were profiled in total and in density fractionated granulocytes by QuantiGene™ Plex assay. The granule biogenesis pathway genes [25] *CEACAM4*, *PLAC8*, and *CD63* were analyzed by quantitative real-time polymerase chain reaction (qRT-PCR) and their abilities to distinguish sepsis and SIRS were compared to the routine infection marker CRP (C-reactive protein). The regulation and sepsis biomarker potential of these genes in patients with stress-induced granulopoiesis are discussed.

## Methods

### Patients

Between June 2020 and January 2022, patients admitted to the interdisciplinary surgical ICU and the surgical IMC of a tertiary care hospital (University Medical Center Mannheim) were screened by chart review for the presence of SIRS and suspected infection [26, 27] related to the reason for admission. Patients qualified for study inclusion within 48 h of admission only if the attending physician confirmed the presence of SIRS or sepsis according to Sepsis-3 [28]. Our main focus was on patients with SIRS and sepsis with comparable disease severity. Patients with septic shock were additionally included as a significantly more severely ill subgroup. The blood lactate levels in septic shock, were required to have exceeded a concentration of 2 mM [28] during the last 24 h. Exclusion criteria were age below 18 years, pregnancy, cardiopulmonary resuscitation, end-stage renal or liver disease, previous organ transplantation, coronavirus disease 2019, and neoadjuvant therapy within the preceding four weeks.

### Workflow overview

**Fig. 1:**
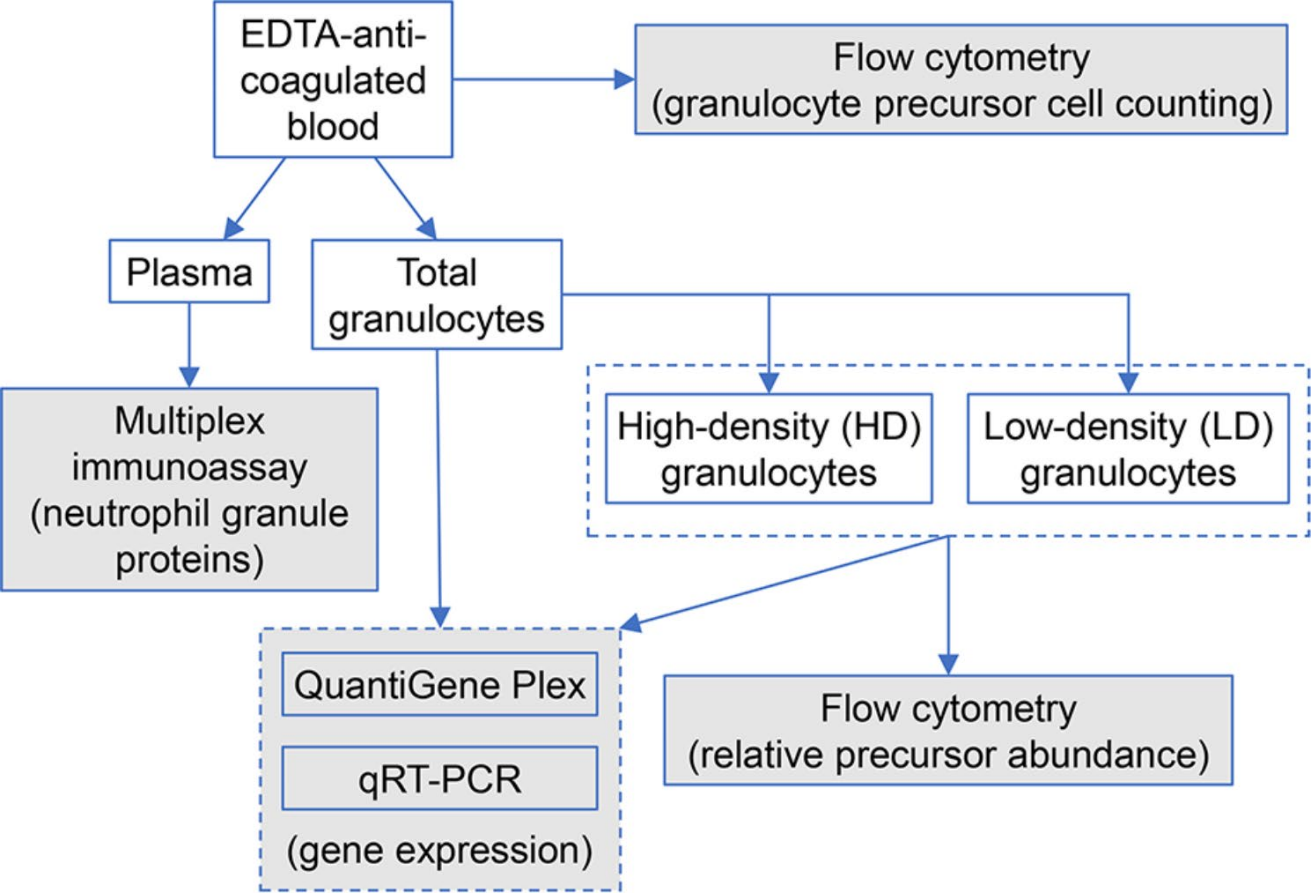
Workflow overview. Blood samples and granulocyte preparations obtained therefrom are indicated in white boxes and analysis methods employed in this study in gray boxes

Blood was collected into S-Monovette^®^K3 EDTA tubes (Sarstedt, Nümbrecht, Germany) from a central venous catheter or an intravenous line, and processed and analyzed as summarized in Fig. 1. Cell counts of granulocyte precursors in whole blood were determined by flow cytometry. From another aliquot of blood, plasma was prepared by centrifugation and stored frozen for multiplex immunoassay analysis. A third aliquot was used for the isolation of total granulocytes on CD15 microbeads, of which one part was retained and another was subjected to density-gradient centrifugation. The relative abundances of precursor populations were profiled in the resultant HD and LD fractions by flow cytometry. QuantiGene™ Plex assays and qRT-PCR were used to profile gene expression in the total, HD, and LD granulocytes. These different analytical approaches defined our analytical subcohorts (Additional File 1: Fig. S1).

### Blood granulocyte isolation and density separation

Total granulocytes were enriched from 12 mL of whole blood by positive selection using StraightFrom™ Whole Blood CD15 MicroBeads according to the manufacturer’s protocol (Miltenyi Biotec). From these, HD and LD granulocytes were prepared by density gradient centrifugation. Five milliliters of total granulocytes were diluted with 5 mL PBS+ (PBS containing 2 mM EDTA) and carefully layered over 3 mL of Ficoll-Paque™ Plus (Cytiva, Marlborough, MA, USA). After centrifugation for 30 min at 400 × g and ambient temperature, the upper layer was removed and the LD granulocyte-containing interphase was transferred to a new tube with 5 mL of PBS+. After removal of the supernatant, the sediment containing the HD granulocytes was resuspended in 500 μL PBS + and transferred to a new tube. HD and LD granulocytes were counted and viabilities were determined by trypan blue staining using a Countess™ II automated cell counter (Thermo Fisher Scientific). Cell viabilities were > 97%.

### Multicolor flow cytometry

Blood counts of granulocyte precursors, i.e., late promyelocytes, myelocytes, metamyelocytes, band cells, and of the mature polymorphonuclear neutrophils were determined by multicolor flow cytometry on a BD FACSLyric™ cytometer using BD Trucount™ tubes (BD Biosciences, San Jose, CA, USA) as described [19]. The reported staining and gating strategies are illustrated in Additional File 1: Fig. S2. Relative granulocyte precursor profiles were determined in HD and LD granulocytes acquiring at least 10^5^ events per run in the CD45^+^ gate, 70–80% of which constituted CD15^+^ cells, i.e., the granulocytic series. FlowJo™ V10 (Tree Star, Ashland, OR, United States) software was used for analysis.

### Multiplex immunoassay

MILLIPLEX^®^ kits (Merck Millipore, Burlington, MA, USA) were used to quantify neutrophil granule proteins in blood plasma on a MAGPIX^®^ system (Luminex Corporation, Austin, TX, United States) according to the manufacturer’s protocols. The Human Sepsis panel 3 (HSP3MAG-63 K) was used to detect elastase 2, lactotransferrin, and neutrophil gelatinase-associated lipocalin (NGAL), and the Human Circulating Cancer Biomarker panel 3 (HCCBP3MAG-58 K) to detect cathepsin D and myeloperoxidase. Duplicate determinations were averaged and analyzed using MILLIPLEX Analyst software (Merck Millipore). Determinations with a coefficient of variation > 20% (10.2% of the data) were treated as missing values and excluded. In four samples, the lactotransferrin levels were above the upper limit of detection, which was used in these cases in the analysis.

### Gene expression analyses

Total, HD, and LD granulocytes were subjected to gene expression analyses. Transcriptional profiles of terminal granulocytic differentiation were determined by branched DNA signal amplification assays (QuantiGene™ Plex). Selected sepsis marker genes were quantified by TaqMan qRT-PCR.

#### QuantiGene Plex™ assay

QuantiGene™ Plex assays (Thermo Fisher Scientific) were run in technical duplicates on a MAGPIX system (Luminex Corporation) as described before [29]. In total, 135 genes, characterizing the different stages of terminal granulocytic differentiation, were multiplexed. *AKIRIN1* served as a reference gene [19, 29]. The GenBank Nucleotide IDs for QuantiGene™ Plex assays and pathway memberships of the genes are given in Additional File 1: Table S1. Determinations below the detection limit (5.6% of the data) were treated as missing values and excluded from the analysis.

#### qRT-PCR analysis

Total RNA was isolated, checked for integrity, quantified, and reverse transcribed as previously described [29]. TaqMan qRT-PCR assays were performed in technical triplicates on a StepOne™ Plus RT PCR System (Applied Biosystems, Thermo Fisher Scientific). Results are presented as CT- and linearized ΔCt-values. TaqMan assays used were Hs01041238_g1 (*CD63*), Hs00930964_g1 (*PLAC8*), and Hs00958102_g1 (*CEACAM4*).

### Data analysis

For comparisons, patients were allocated to the three subgroups SIRS, sepsis, and septic shock. Clinical characteristics of subgroups were compared with the t-test (Satterthwaite method) for continuous parameters and with the Chi² test or Fisher’s exact test for categorical parameters using SAS V9.4 (Statistical Analysis System, SAS Institute, Cary, NC, USA). The results from our analyses of total, HD and LD granulocytes were compared with Prism 9 (GraphPad Software, San Diego, CA, USA). Pairwise comparisons of dependent and independent samples were done with the Wilcoxon signed-rank test and the Mann-Whitney U test, respectively. Multiple independent samples were compared with the Kruskal-Wallis test using Dunn’s test for post hoc pairwise comparisons. We used Spearman’s rank-order correlation to assess correlations between SOFA scores and blood counts of granulocyte precursors populations. Spearman’s ρ was interpreted according to Dancey and Reidy (2004) [30]. In all analyses, *p*-values of < 0,05 were considered statistically significant.

## Results

### Patient characteristics

The patient characteristics on study inclusion, hospital length of stay (LOS), and hospital mortality as well as results of statistical patient subgroup comparisons are tabulated for the total cohort and each analytical subcohort in Additional File 1: Tables S2–S8. The patient-level information on subcohort membership, SIRS etiology (type of surgery), infectious focus and agent as well as the SOFA score is available from heiDATA (https://doi.org/10.11588/data/JRWPFV).

Sixty patients in total were included: 24 SIRS (of which 21 were post-surgical), 25 with sepsis, and 11 with septic shock. A detailed description of the patient characteristics for the total cohort is given in Additional File 1: Text S1. Briefly, the average (standard deviation) age was 65.4 (12.9) years. 40% were female. General surgery was the most frequently admitting department (55.0%), and the mean hospital LOS was 24.6 (22.5) days. There were no statistically significant subgroup differences across the total cohort in demographics, admitting department, and hospital LOS. As expected, patients with septic shock had higher disease severity as measured by the SOFA score (median SOFA = 10) and hospital mortality (81.8%) than those with SIRS (median SOFA = 4, hospital mortality = 12.5%) and sepsis (median SOFA = 4, hospital mortality = 16.0%). Tumor disease was more frequent in SIRS (62.5%), where it was a common reason for surgery, than in sepsis (28.0%) and septic shock (18.2%). Higher levels of the inflammatory blood markers WBC (white blood cell) count and CRP in the latter two subgroups compared to SIRS agreed with the presence of infection. Noteworthily, 1.43-fold higher mean WBC counts [18.0 (9.86) versus 12.6 (3.36) × 10^9^/L, *p* = 0.0154] and 2.18-fold higher mean levels of CRP [211 (99.8) versus 96.8 (62.5) mg/L, *p* < 0.0001] were the only statistically significant differences between sepsis and SIRS besides more frequent tumor disease in SIRS.

The clinical characteristics of our three patient subgroups in the total cohort and each of the analytical subcohorts are juxtaposed as radar charts in Additional File 1: Fig. S3. In all subcohorts, septic shock remained associated with the worst values (Additional File 1: Tables S2–S8). The following summary of clinical characteristics in the subcohorts thus focuses on the comparison of sepsis and SIRS. In the 51- and 42-patient flow cytometry subcohorts used to determine precursor blood counts and the relative subpopulation abundance, respectively, in high- and low-density granulocyte fractions, subgroup differences (Additional File 1: Tables S3 and S4) were highly similar to those in the total cohort. For both subcohorts, a higher mean value for CRP in sepsis than SIRS was the only statistically significant difference between these two subgroups. This difference was also seen in the 53-patient multiplex immunoassay subcohort used to determine granule proteins in plasma. In this subcohort, the frequency of tumor disease and the mean WBC count were higher and lower, respectively, in SIRS than sepsis, as in the total cohort, and the mean blood pH was lower in sepsis than SIRS (Additional File 1: Table S5). Despite high relative variations in the small 15-patient QuantiGene™ Plex subcohort with only five patients per subgroup, only a lower mean blood pH in sepsis reached statistical significance (Additional File 1: Table S6) as in the multiplex subcohort. In both the 30-patient qRT-PCR subcohort used to analyze the total granulocytes as well as in the 25-patient qRT-PCR subcohort used for the HD and LD granulocytes, fully contained in the first qRT-PCR subcohort, only higher mean CRP values in sepsis than SIRS were statistically significant. Notably, the *p*-values for the sepsis versus SIRS comparison of the SOFA scores ranged between 0.29 for the qRT-PCR (HD and LD granulocytes) subcohort and 1.00 for the QuantiGene™ Plex subcohort. This argues against important differences in disease severities between patients with sepsis and SIRS across our analytical subcohorts.

### Granulocyte precursor blood counts

**Fig. 2:**
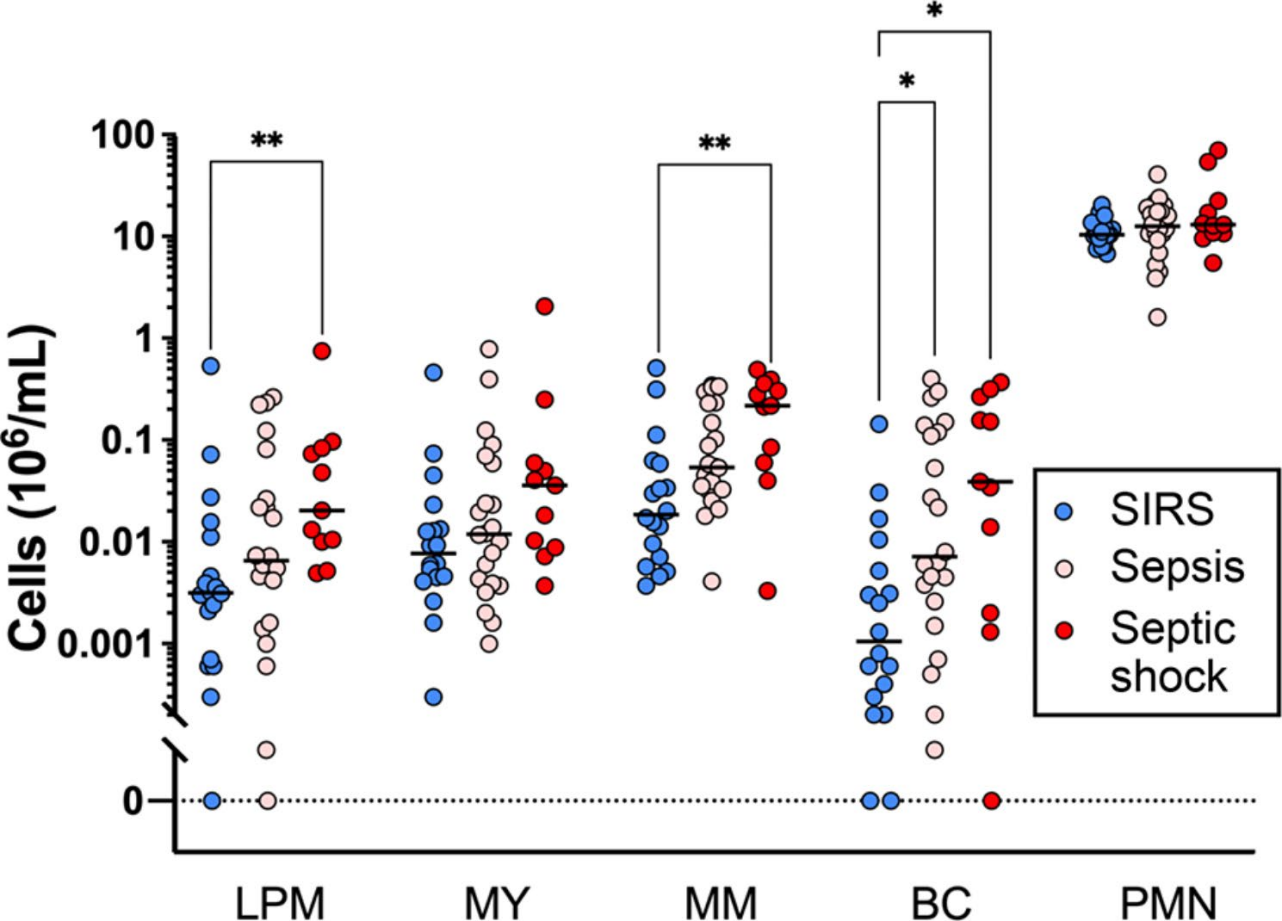
Blood counts of granulocyte precursors. Flow cytometric determination of cell counts for stages of terminal granulocytic differentiation in SIRS (*n* = 18), sepsis (*n* = 22), and septic shock (*n* = 11). Absolute cell numbers per blood volume were determined using BD Trucount beads. Median counts are indicated by solid horizontal lines. LPM: late promyelocyte; MY: myelocyte; MM: metamyelocyte; BC: band cell; PMN: polymorphonuclear neutrophil. * *p* < 0.05; ** *p* < 0.01, from the Kruskal-Wallis test with Dunn’s post hoc test

Across all precursor populations and the polymorphonuclear neutrophils (PMNs), blood counts were highest in the septic shock and lowest in the SIRS patients (Fig. 2). The count difference between the two patient subgroups reached statistical significance for late promyelocytes, metamyelocytes, and band cells with median differences of 6.5-fold, 37.8-fold and 11.7-fold, respectively. For the intermediate sepsis subgroup, only a 2.9-fold higher median band cell count compared to the SIRS patients reached statistical significance. These results are consistent with previous reports of higher granulocyte precursor blood counts in sepsis than SIRS on ICU admission [19, 31].

Next, we assessed whether our granulocyte precursor counts correlated with disease severity measured by the concurrent SOFA score (Additional File 1: Fig. S4). Correlations were only observed for band cells (*p* = 0,0024, ρ = 0,513) and metamyelocytes (*p* = 0,0267, ρ = 0,386) when the septic shock and sepsis subgroups were combined. They persisted when the SIRS subgroup was added. However, counts of neither the most mature (PMNs) nor least mature population (late promyelocytes myelocytes) showed any correlation with SOFA.

### Relative abundance of precursor and mature granulocytes in the HD and LD fractions

**Fig. 3:**
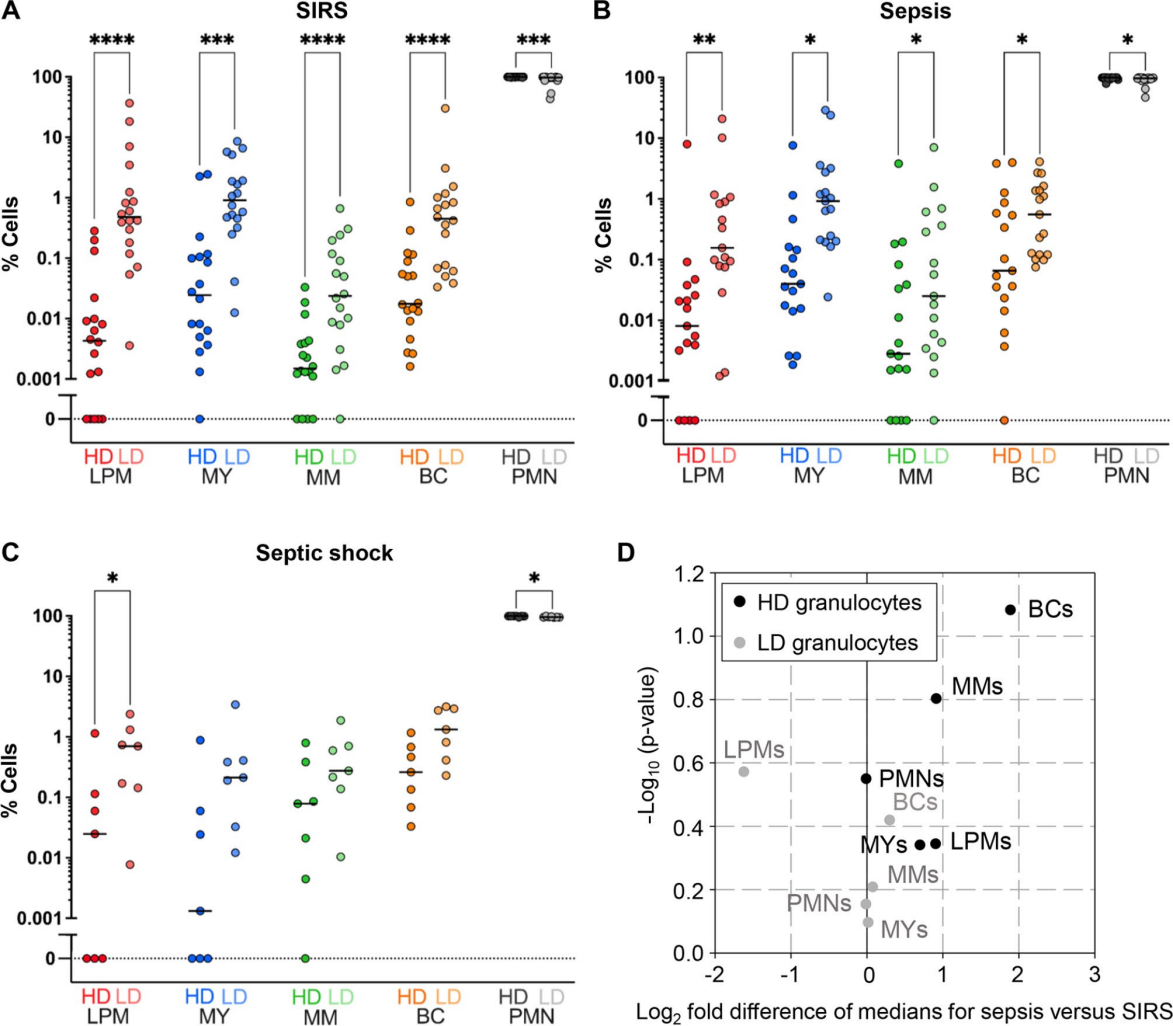
Relative abundance of granulocyte precursors in the low- and high-density fractions. The proportions of granulocyte precursors and mature cells within the high-density (HD) and the low-density (LD) fractions were determined by flow cytometry in patients with (**A**) SIRS (*n* = 18), (**B**) sepsis (*n* = 17) and (**C**) septic shock (*n* = 7). Median proportions are indicated by solid horizontal lines. (**D**) The median differences of the cell proportions in sepsis compared to SIRS are plotted against their statistical significance. LPM: late promyelocyte; MY: myelocyte; MM: metamyelocyte; BC: band cell; PMN: polymorphonuclear neutrophil. * *p* < 0.05; ** *p* < 0.01, *** *p* < 0.001, **** *p* < 0.0001, from the Wilcoxon signed-rank test for (A–C) and from the Mann-Whitney U test for (D)

The median proportions of the granulocyte precursor subpopulations were generally higher in the LD than HD fractions across all patient subgroups (Fig. 3A–C) consistent with their expected enrichment in the LD fraction. For all precursors, the difference was overall most pronounced in SIRS, reaching 111-fold for late promyelocytes (Fig. 3A), followed by sepsis (Fig. 3B) and septic shock (Fig. 3C). Due to the low patient number in the latter subgroup, statistical significance was reached only for late promyelocytes. In all subgroups, mature PMNs were still by far superior in counts in either fraction, ranging from a median of 95.4% in the LD fraction from septic shock patients to 99.8% in the HD fraction from SIRS patients. PMNs did not appear depleted in the LD fraction. Nevertheless, 2%, 3%, and 4% lower proportions in the LD than the HD fractions in SIRS, sepsis, and septic shock, respectively, all reached statistical significance.

There were no differences between sepsis and SIRS in relative abundance for any granulocyte maturation stage in the LD or HD fraction (Fig. 3D).

### Neutrophil granule proteins in blood plasma

**Fig. 4:**
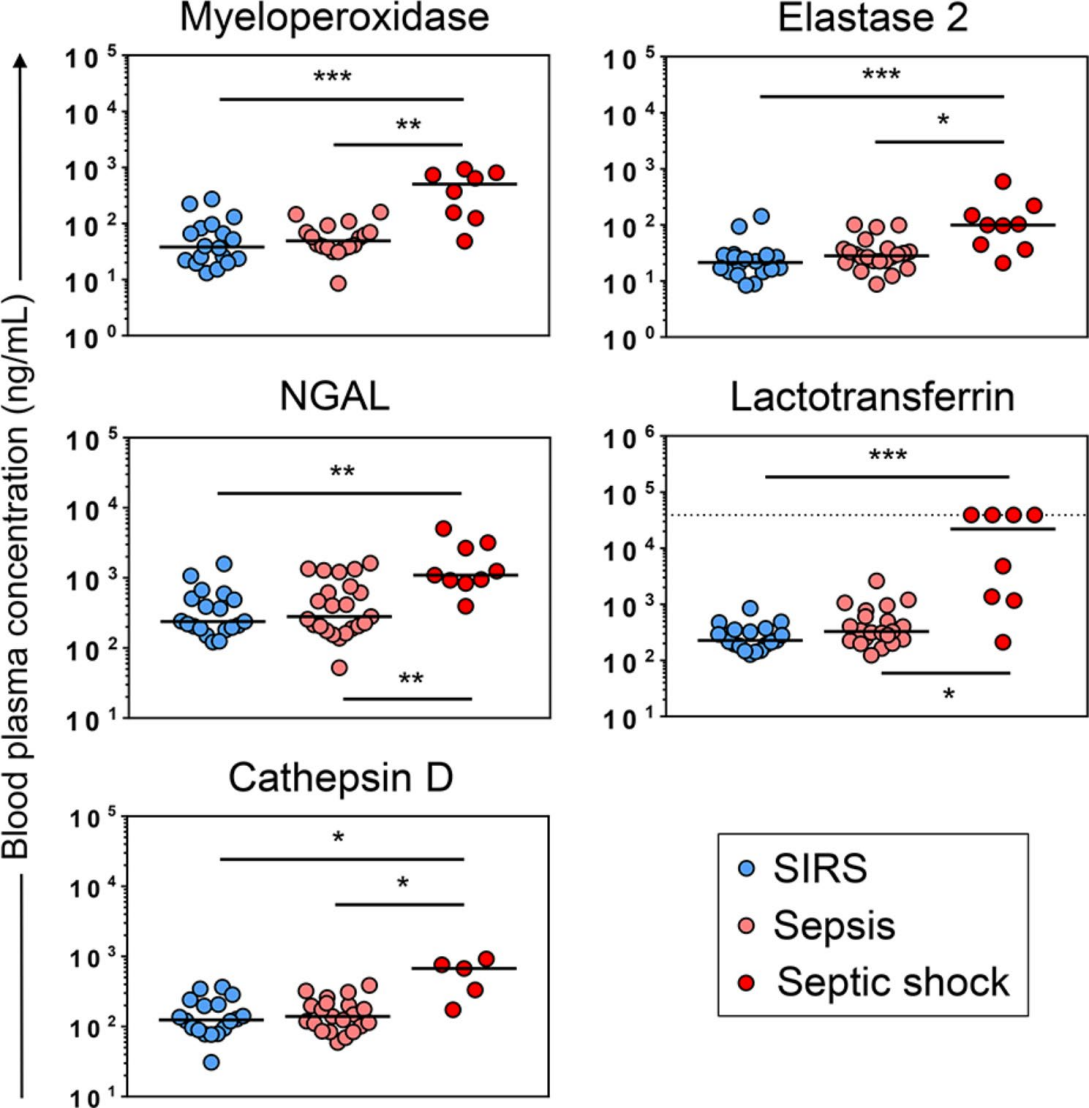
Blood plasma levels of neutrophil granule proteins in Sepsis and SIRS. Plasma proteins were multiplexed in patients with SIRS (*n* = 21), sepsis (*n* = 23), and septic shock (*n* = 9). Solid horizontal lines indicate median concentrations. The horizontal dotted line with lactoferrin indicates the upper limit of detection. NGAL: neutrophil gelatinase-associated lipocalin. * *p* < 0.05; ** *p* < 0.01, *** *p* < 0.001, from the Kruskal-Wallis test with Dunn’s post hoc test

Blood plasma levels of the azurophilic granule enzyme myeloperoxidase were previously shown to be elevated in sepsis and especially septic shock compared to SIRS [32] suggesting a higher degree of granulocyte degranulation in sepsis than SIRS. Degranulation is expected to lower the buoyant density of granulocytes, potentially leading to a shift from the HD to the LD fraction [33]. Therefore, we determined the plasma levels of myeloperoxidase and four additional prototypical neutrophil granule proteins: elastase 2 (azurophilic granules), NGAL and lactotransferrin (both specific granules), and cathepsin D (ficolin-containing granules) (Fig. 4).

Throughout, the median plasma levels of all five proteins were significantly higher in septic shock than in both sepsis and SIRS. They were, however, only marginally higher in sepsis than SIRS, ranging from only 1.1-fold for cathepsin D to 1.5-fold for lactotransferrin, not reaching statistical significance in any case. This supports more blood granulocyte degranulation in septic shock than in sepsis and SIRS without an important difference, however, between the latter two.

### Transcriptional profiling of granulocytic differentiation in total, LD, and HD granulocytes by QuantiGene™ Plex analysis

According to our previous results, gene signatures of early terminal granulocytic differentiation, determined in total whole blood granulocytes, distinguish sepsis from SIRS on ICU admission [19]. Because all granulocyte precursor populations were enriched in the LD over the HD fraction (Fig. 3), we predicted that the sepsis-SIRS contrast in these gene signatures was more pronounced in the LD fraction and less in the HD fraction each compared to total granulocytes. To test this, we assembled a panel of signature genes that characterize the granulocytic series from promyelocytes to PMNs. We applied the following rules to select these genes based on the results of our previous transcriptome analysis [19]. First, for each of the seven canonical promyelocyte- and myelocyte-restricted pathways showing the strongest enrichment in sepsis compared to SIRS (i.e., OxPhos, Proteasome, Ribosome, Lysosome, Cell Cycle, TCA Cycle, and Fatty Acid Metabolism), the ten genes with the highest running enrichment scores were included. We additionally included here the sepsis-associated Carbon Metabolism pathway [19]. Second, signature genes of granule biogenesis pathways, differentially expressed in sepsis and SIRS as reported [19], were included if the log_2_ mean difference for sepsis versus SIRS was > 0.4. The pathways comprised Azurophilic Granules (promyelocytes), Specific Granules (myelocytes), Gelatinase-Containing Granules (metamyelocytes), Ficolin-Containing Granules (band cells), Secretory Vesicles (segmented neutrophils), and Cell Membrane (PMNs). Third, eight differentially expressed lysosomal/endolysosomal genes were included, for which we had independently validated higher expression in sepsis than in SIRS CD15^+^ cells [19]. An overview of the resultant 135-gene QuantiGene™ Plex test panel with pathway memberships is given in Additional file 1: Table S1. Total granulocytes and the derived HD and LD fractions from five patients each with SIRS, sepsis, and septic shock were subjected to QuantiGene™ Plex analysis.

#### Comparisons of total, HD, and LD granulocytes

**Fig. 5:**
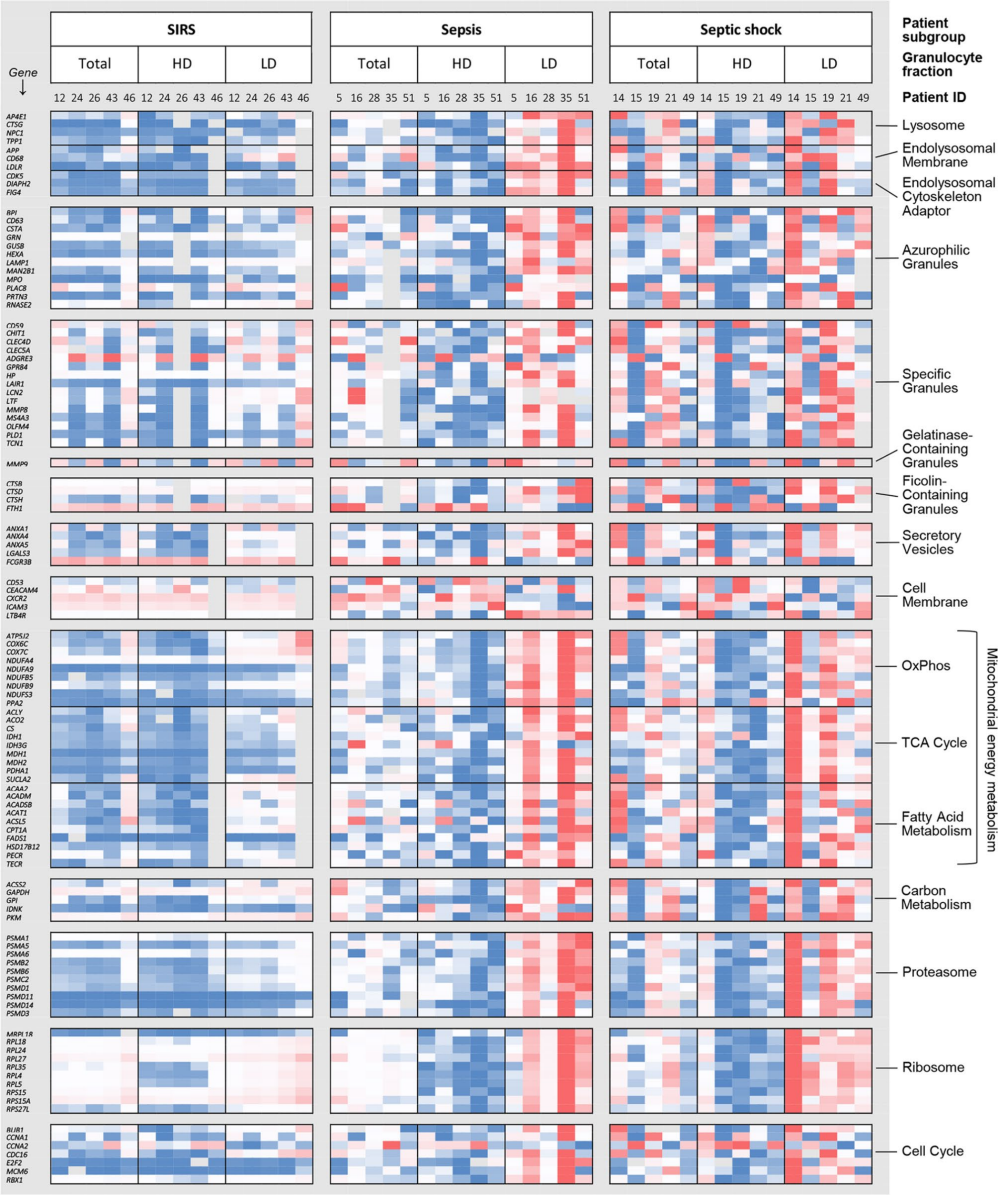
Heat map of QuantiGene™ Plex results for granulocyte test panel genes. Genes with a *p*-value of 0.0625 from the Wilcoxon signed-rank test, the smallest observed value, in any of the three pairwise comparisons of the CD15 fractions, i.e., the total, high-density (HD), and low-density (LD) fractions, are arranged by pathway. Pathway memberships are indicated to the right. In cases of dual pathway memberships, the label for the pathway accounting for inclusion into the analysis was used and, where applicable, labels for granule biogenesis pathways were given priority. Per row, expression levels are min (blue)-max (red)-scaled. Grey tiles indicate missing values

We conducted pairwise comparisons of the obtained results for the total, HD, and LD granulocyte preparations. Due to the low number of five patients per subgroup, the lowest *p*-value obtained in any comparison was 0.0625, which applied to altogether 112 genes (83% of the test panel) summarized in Fig. 5.

Overall, the expression levels were most similar across all cell preparations and patients within the SIRS subgroup and showed the highest inter-patient variations in septic shock. In SIRS compared to sepsis HD granulocytes, the relatively high expression levels of the genes encoding the ribosomal proteins L18, L24, L27, S15, and S15A stood out. However, none of the expected enrichments of early granule biogenesis and promyelocyte- and myelocyte-restricted canonical pathways in the LD fraction was apparent in SIRS other than partially for Fatty Acid Metabolism and OxPhos. In sepsis contrarily, the expression profile overall agreed with our previous results [19]. Namely, the levels for the selected endolysosomal, granule biogenesis except for the Cell Membrane pathway, and canonical pathway genes were highest in the LD and lowest in the HD fractions. The intermediate position of the total granulocyte preparation in sepsis appeared clearest for the Ribosome pathway. This data indicates that the higher expression of signature genes of early terminal granulocytic differentiation in sepsis than SIRS was more pronounced in the LD and less in the HD fraction compared to the parent total granulocyte preparation.

#### Comparisons of patient subgroups

**Fig. 6:**
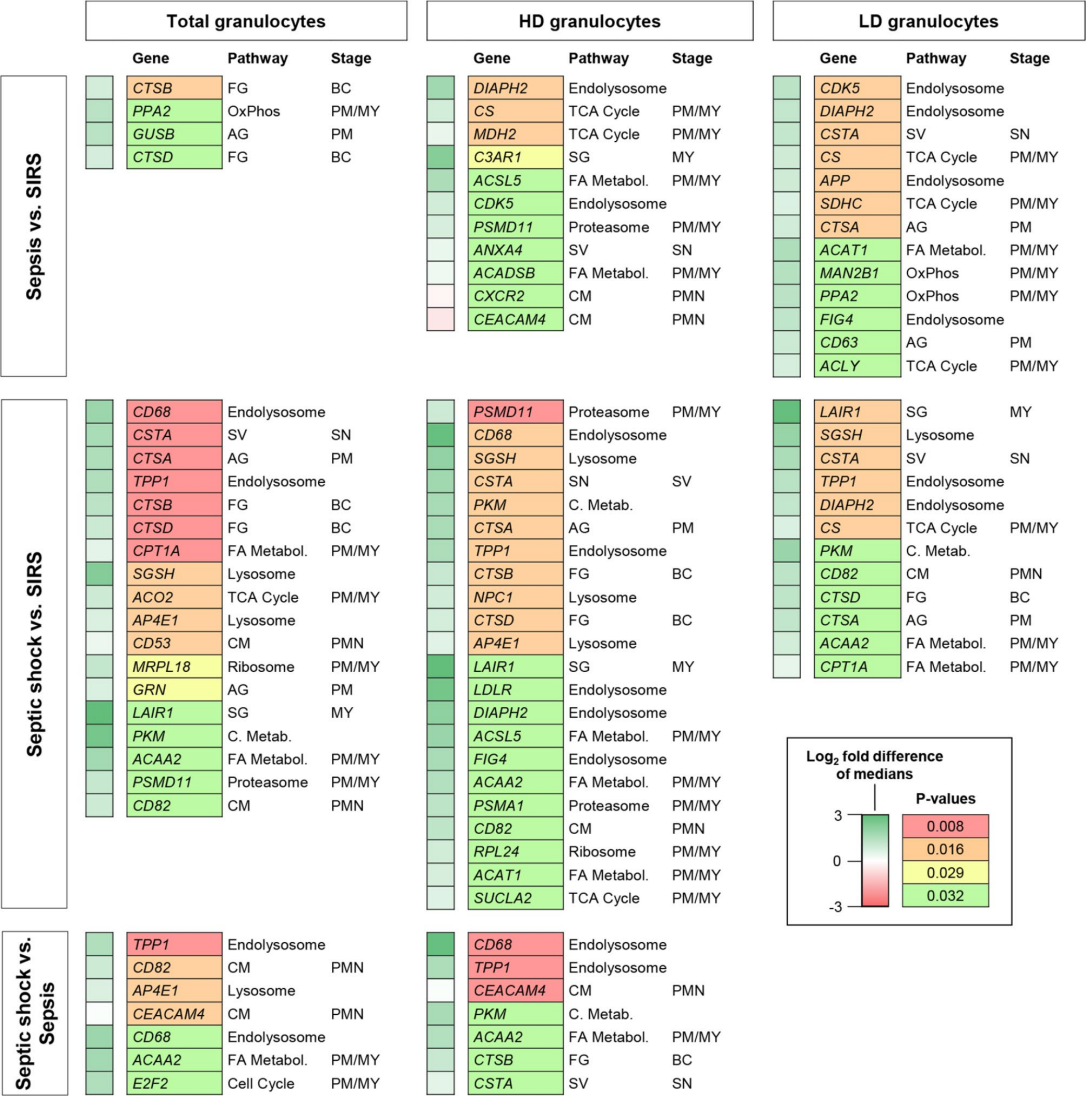
Statistically significant patient subgroup differences in gene expression by the QuantiGene™ Plex assay for total, high-density (HD), and low-density (LD) granulocytes. Median fold differences and *p*-values from the Mann-Whitney U test are color-coded as indicated in the legend at the bottom right. Pathway memberships accounting for inclusion into the test panel and corresponding granulocyte precursor stages are identified. Granule biogenesis pathway abbreviations are AG: Azurophilic Granules; SG: Specific Granules, FG: Ficolin-Containing Granules; SV. Secretory vesicles; CM: Cell Membrane. Canonical pathway abbreviations are C. Metabol.: Carbon Metabolism; FA Metabol.: Fatty Acid Metabolism. Precursor abbreviations are PM: promyelocyte; MY: myelocyte; BC: band cell; PMN: polymorphonuclear neutrophil

Next, we considered the pairwise comparisons of gene expression in total, HD and LD granulocytes between the three patient subgroups (Fig. 6 and Additional File 1: Fig. S5). With two exceptions, the expression levels for altogether 42 differentially expressed genes (DEGs) (31.1% of the test panel) across all subgroup comparisons were lower in SIRS than in sepsis or septic shock, and lower in sepsis than in septic shock. For sepsis versus SIRS and septic shock versus SIRS, we counted 24 and 28 DEGs, respectively, distributed over all three granulocyte preparations in each comparison. For sepsis versus SIRS, there was no overlap in the DEGs between total and HD granulocytes, and little overlap between LD and total as well as LD and HD granulocytes (Additional File 1: Fig. S5A). Contrarily, 46.4% of the DEGs from the septic shock versus SIRS comparison were shared between total and HD granulocytes, and 32.1% between all three preparations (Additional File 1: Fig. S5B). In the comparison of septic shock versus sepsis, there were merely seven DEGs each in the total and the HD granulocytes with four shared between these two preparations (Additional File 1: Fig. S5C).

Promyelocyte- and myelocyte-restricted canonical pathways were represented among the DEGs across all comparisons and granulocyte preparations (Fig. 6). Notably, the only granule biogenesis pathway genes, known to be restricted to these two earliest precursor stages [25] and indeed represented in the LD granulocytes, were *CTSA* and *CD63* (both Azurophilic Granules) in the sepsis versus SIRS comparison, and *CTSA* and *LAIR1* (the latter Specific Granules) in the septic shock versus SIRS comparison. As the above-mentioned exception, the two PMN-expressed Cell Membrane pathway genes, *CXCR2* and *CEACAM4*, showed higher expression levels in HD granulocytes from patients with SIRS than sepsis. Given the absence of any differences between sepsis and SIRS in PMN blood counts (Fig. 2) and their proportions in both HD and LD granulocytes (Fig. 3D), PMNs appear to express lower levels of *CXCR2* and *CEACAM4* in sepsis than SIRS.

#### Differential expression profiles of SeptiCyte™ LAB genes and CD63 prompt their selection for RT-PCR validation

We noted that all four genes of the SeptiCyte™ LAB test [11, 12] had been selected as bona fide granule biogenesis pathway genes [25] in our 135-gene QuantiGene™ Plex test panel (Additional File 1: Table S1). These were the promyelocyte-restricted Azurophilic Granule pathway genes *LAMP1* and *PLAC8* as well as the PMN-restricted Cell Membrane pathway genes *CEACAM4* and *PLA2G7*. *LAMP1* and *PLAC8* both appeared more highly expressed in LD than total granulocytes of SIRS patients and in LD than HD granulocytes in sepsis patients (Fig. 5), consistent with granulocyte precursors as their source, but no patient subgroup differences were seen for either gene (Fig. 6). The latter was most likely due to the limited number of five patients per subgroup. *CEACAM4*, however, appeared not only more highly expressed in total and HD granulocytes than in the LD fraction of septic shock patients (Fig. 5), consistent with PMNs as their source, but also showed higher levels in SIRS than sepsis HD granulocytes (Fig. 6). The latter led us to select *CEACAM4* for validation by qRT-PCR in HD and total granulocytes. Because the SeptiCyte™ LAB score is based on the expression level ratio of the Azurophilic Granule to the Cell Membrane pathway genes [11], we further included one of the former, *PLAC8*. Additionally, we selected the Azurophilic Granule gene *CD63* [34, 35] because its levels were exclusively higher in sepsis than SIRS LD granulocytes, which would also have been our expectation for *PLAC8* and *LAMP1*. We hypothesized that forming the expression level ratio of each *PLAC8* and *CD63* from LD granulocytes to *CEACAM4* from the HD fraction improves the discrimination between sepsis and SIRS compared to the LD levels of these genes considered separately.

### Elevated expression of *CD63* but not *PLAC8* in sepsis is restricted to LD granulocytes

**Fig. 7:**
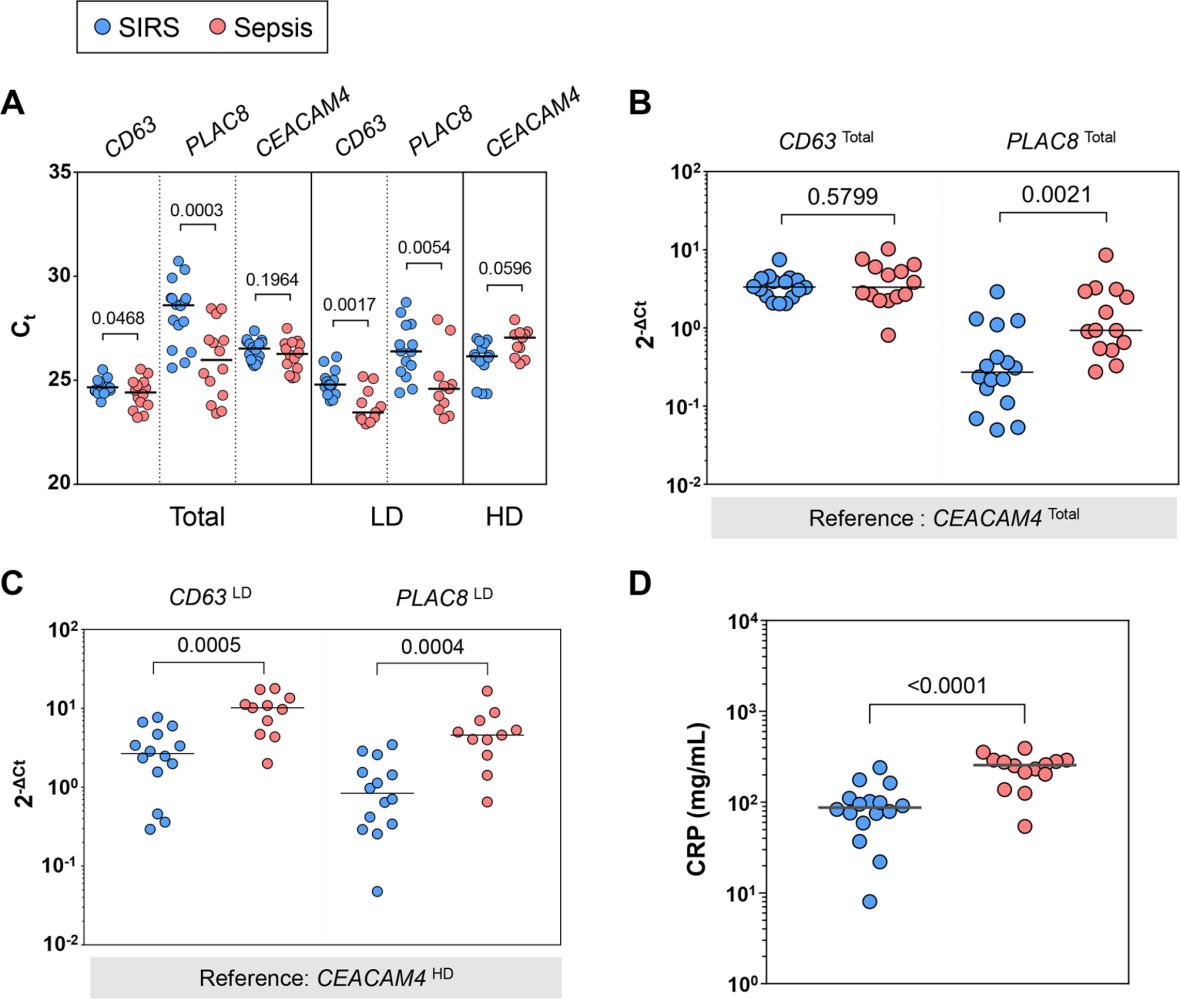
Quantitation of *CD63*, *PLAC8*, and *CEACAM4* expression in the granulocyte fractions and C-reactive protein (CRP) in plasma. Patient subgroup medians are represented by horizontal lines. Superscripts to the gene names identify granulocyte preparations. Total granulocytes from 14 patients with sepsis and 16 with SIRS were analyzed by qRT-PCR. Accordingly, high-density (HD) and low-density (LD) granulocytes from 11 patients with sepsis and 14 with SIRS were analyzed. Panel (**A**) summarizes the cycle threshold (C_t_) values for all measurements. Total granulocyte levels of *CD63* and *PLAC8* were referenced to *CEACAM4* from the total granulocytes (**B**) and LD granulocyte levels to *CEACAM4* from the HD fraction (**C**). Routine lab values for CRP are shown for the same patients contributing total granulocytes to the QRT-PCR analysis (**D**). The *p*-values from the Mann-Whitney U test are indicated

The distributions of the qRT-PCR data are displayed in Fig. 7A–C. Higher expression levels of *CD63* and *PLAC8* in sepsis than SIRS reached statistical significance in both total and LD granulocytes. This difference was most pronounced for *PLAC8* and only marginal for CD63 in the total granulocytes. After referencing to *CEACAM4*, the difference in total granulocytes persisted for *PLAC8* but not *CD63* (Fig. 7B). In the LD granulocytes by contrast, the sepsis-SIRS differences in *CD63* and *PLAC8* expression were comparable (Fig. 7C). As hypothesized above, this difference was enhanced for both genes after referencing the measurements from the LD fractions to *CEACAM4* determined in the corresponding HD fractions. Taken together, this indicates that elevated expression of *CD63* but not *PLAC8* in sepsis is restricted to LD granulocytes and that a concomitant reduction of *CEACAM4* expression in HD granulocytes improves the ratiometric discrimination between sepsis and SIRS.

Among the clinical characteristics, a higher blood value of the infection marker CRP in patients with sepsis than SIRS was the only significant patient subgroup difference in the qRT-PCR subcohort (Additional File 1: Table S7). The median CRP concentration in sepsis was 2.94-fold higher than in SIRS (Fig. 7D). This was only exceeded by a 6.20-fold higher median level of *PLAC8* expression in total granulocytes (Fig. 7A).

## Discussion

Emergency granulopoiesis in patients admitted to the ICU with sepsis compared to SIRS was associated with higher blood counts of immature granulocyte populations in this (Fig. 2) and previous studies [19, 31]. This led us to hypothesize that the corresponding increase in the activities of promyelocyte- and myelocyte-restricted transcriptional pathways in sepsis [19] was attributable to elevated counts of LD rather than HD granulocytes in which the immature and mature granulocytes are enriched, respectively [24]. The proportions of each precursor population were indeed at least one order of magnitude higher in the LD than the HD fraction (Fig. 3A, B). However, there were no sepsis-SIRS differences in the corresponding HD and LD proportions for any granulocytic differentiation stage (Fig. 3D). This indicates that the absolute and not the proportionate increase in immature granulocytes accounts for the potential of transcriptional pathways of early terminal granulocytic differentiation to distinguish sepsis from SIRS, and thus confirms our hypothesis. These pathways also appeared intrinsically more active in LD granulocytes of patients with sepsis (Fig. 5) further adding to their discriminatory performance. Similarly, a recent single-cell RNA-sequencing (scRNA-seq) analysis demonstrated supra-physiological and partly untimely and prolonged expression of early developmental marker genes in immature granulocytes from healthy donors treated with granulocyte colony-stimulating factor (G-CSF) and in patients after transplantation of hematopoietic stem cells (HSCs-T) [36]. The elevations were particularly pronounced for several marker genes of early granule biogenesis.

HD and LD granulocytes displayed distinct transcriptional sepsis-SIRS differences with eight DEGs in the HD, nine in the LD fraction, and only three shared (Fig. 6, Additional File 1: Fig. S5A). In the septic shock-versus-SIRS comparison by contrast, ten out of the twelve DEGs in LD granulocytes were shared with the HD fraction which featured twelve additional DEGs (Fig. 6 and Additional File 1: Fig. S5B). This emphasizes the value of considering both fractions separately especially when comparing sepsis and SIRS. Particularly, HD granulocytes showed higher levels of the PMN-expressed Cell Membrane pathway genes *CXCR2* and *CEACAM4* in SIRS than sepsis. For *CXCR2*, this agrees with reportedly reduced sepsis levels of the encoded chemokine receptor at the cell surface [37, 38], which are also due to activation-induced receptor internalization. Reduced levels of *CEACAM4*, encoding a phagocytosis-mediating receptor [39], in whole blood of sepsis patients are integral to the SeptiCyte™ LAB test [11]. However, the lower *CEACAM4* levels in sepsis than SIRS did not reach statistical significance in either total or HD granulocytes when determined by qRT-PCR (Fig. 7A). But using the levels of *CEACAM4* in the HD fraction as a reference for *PLAC8*, also a SeptiCyte™ LAB gene, and *CD63* in the LD fraction still improved the sepsis-SIRS distinction by these two azurophilic granule genes compared to their unreferenced LD levels (Fig. 7C). Interestingly, unreferenced *PLAC8* showed the largest and *CD63* the smallest sepsis-SIRS difference in total granulocytes, whereas for both genes the sepsis-SIRS differences in the LD fractions were comparable (Fig. 7A). This indicates that, as expected, the increased sepsis levels of *CD63* compared to SIRS largely stem from immature granulocytes but that *PLAC8* expression is additionally increased in PMNs. In the above-mentioned scRNA-seq analysis by Montaldo at el. (2022), G-CSF treatment and HSC-T approximately doubled *PLAC8* expression at the promyelocyte stage [36]. G-CSF induced a further increase of *PLAC8* during differentiation and sustained promyelocyte-like levels in mature granulocytes. We assume, that sepsis induces a similar *PLAC8* dynamics during granulopoiesis as G-CSF.

The difference in the regulation of *CD63* and *PLAC8* may be related to the different biological roles that the respective encoded proteins play in granulocytes. The tetraspanin family member CD63 antigen routes the elastase 2 precursor from the ER/Golgi to azurophilic granules through direct interaction [35]. In sepsis patients [40] and upon neutrophil activation in vitro [34], CD63 becomes detectable at the cell surface. Placenta-specific gene 8 protein (PLAC8) functions as a transcriptional regulator [41] and, as a lysosomal membrane-associated protein, regulates autophagolysosomal homoeostasis [42, 43]. In contrast to CD63, PLAC8’s specific function in granulocytes is unknown. One of the multiple activities of *PLAC8* may account for differences in the control of its developmental and potentially also inducible expression compared to *CD63*. This may explain elevated and prolonged activation of *PLAC8* up to the PMN stage in sepsis, while the elevated sepsis levels of *CD63*, encoding a mediator of protein trafficking to azurophilic granules, remains restricted to immature granulocytes.

Sepsis gene signatures in whole blood are known to correlate with disease severity and mortality [6, 14], and our previous identification of genes and pathways of early terminal granulocytic differentiation as markers of sepsis on ICU admission [19] included cases with significantly higher values for SOFA than in the controls. The SOFA score assesses organ dysfunction and predicts mortality on ICU admission [44]. To avoid disease severity as a driver of transcriptional differences in the present study, we enrolled sepsis and SIRS patients with similar disease severities and no subgroup difference in SOFA (Additional File 1: Tables S2–S8). Noteworthily, no significant confounding effect of disease severity, measured among others by SOFA, on the discriminatory performance of the SeptiCyte™ Lab test was described in the combined original discovery and validation cohorts [11]. This agrees with our observation that there were no significant correlations between SOFA and granulocyte blood counts in the SIRS and sepsis patients (Additional File 1: Fig. S4). When, however, the septic shock subgroup was included in the correlation analysis, counts of metamyelocytes and band cells, respectively, showed moderate and strong positive correlations with SOFA while those of late promyelocytes, myelocytes and PMNs showed none. Because promyelocytes express *PLAC8* and PMNs *CEACAM4*, and potentially sustain high levels of *PLAC8*, the expression of these genes may be expected to not increase further with disease severity, as in septic shock, as a result of elevated precursor counts. Nevertheless, they may still increase in this situation due to further cell intrinsic expression activation. Hence, we note that extreme disease severity may still confound the performance of these genes as selective biomarkers of infection.

Based on area under the curve-values from receiver operating characteristic analyses, the SeptiCyte™ Lab test reportedly outperformed the infection marker procalcitonin (PCT) [11, 12, 45] but, conspicuously, not CRP in discriminating sepsis from SIRS [11]. This was originally explained with selection bias as the attending physicians had retrospectively assessed infection taking CRP into account [11]. However, we would expect PCT to similarly guide the clinical diagnosis of infection. Moreover, CRP also showed better performance than PCT and another whole blood host response gene signature developed to distinguish sepsis from SIRS and uncomplicated infection [46]. In the current study, *PLAC8* expression in total granulocytes rivaled the discriminatory performance of CRP, showing a twofold larger increase in sepsis than CRP (Fig. 7A, D).

Despite at least ten-fold enrichments of all immature granulocyte stages in the LD over the HD fractions in all patient subgroups, it is noteworthy that over 95% of cells in the LD fractions were still classified as PMNs by flow cytometry (Fig. 3). Partial degranulation of PMNs in sepsis compared to healthy controls was recently reported to decrease neutrophil buoyant density leading to their shift to the LD fraction [47]. Moreover, Neutrophil Activation was described as the most significantly and strongly enriched Gene Ontology category in the whole blood transcriptomes of postoperative patients over the three days preceding the clinical diagnosis of infection compared to matched postoperative infection-free controls [48]. Against this background and given the similar degranulation marker plasma levels in sepsis and SIRS (Fig. 4), we assume that degranulation contributed to the similarly high proportions of PMNs in the LD granulocytes from our sepsis as well as SIRS patients.

Noteworthily, the flow cytometry protocol that we used to differentiate granulocyte differentiation stages in blood was originally established in blood from G-CSF treated healthy donors [49]. On the same note, the observation that the activities of certain canonical and granule biogenesis transcriptional pathways are restricted to specific stages of the granulocytic series, which we refer to in our current and previous studies [19], was originally made in normal bone marrow [25, 50]. We argue that normal and stress-induced granulopoiesis in sepsis, post-traumatic SIRS, G-CSF treated, and HSC-T patients may follow variable dynamics with regard to transcriptional programs, occurrence of population markers at the cell surface, nuclear morphology, granule composition, and eventually cellular buoyant density. Consequently, the relations among these dimensions along the developmental trajectory may vary in granulopoiesis induced by different stress stimuli.

In conclusion, elevated activity of transcriptional programs of early terminal granulocytic differentiation in blood distinguishes sepsis from SIRS due to higher counts of immature granulocytes in sepsis. Elevated activity of early developmental genes further adds to their discriminatory performance. In the case of the azurophilic granule gene *PLAC8*, but not *CD63*, this elevation remains sustained up to the PMN stage. This likely accounts for the selection of *PLAC8* in the whole blood SeptiCyte™ LAB and the simplified SeptiCyte™ RAPID tests [51]. *PLAC8* in total granulocytes rivals CRP as a blood biomarker of sepsis. Future studies should clarify whether infection-specific transcriptional pathways of granulocytic differentiation exist that differentiate sepsis from other causes of stress-induced granulocytosis more reliably than CRP.

## Electronic supplementary material

Below is the link to the electronic supplementary material.

Supplementary Material 1

## Data Availability

Patient-level data on the clinical patient characteristics considered in this study are not publicly available due to patient privacy but are available from the corresponding author upon reasonable request. Information on patient subcohort memberships, SIRS etiologies, septic focus, infectious agent, flow cytometry, multiplex immunoassay, and QuantiGeneTM Plex, and qRT-PCR results as well as CRP-values are available from heiDATA (https://doi.org/10.11588/data/JRWPFV).

## Abbreviations

CRP: C-reactive protein
DEG: Differentially expressed gene
G-CSF: Granulocyte colony-stimulating factor
HD: High-density
HSCs-T: Hematopoietic stem cells
ICU: Intensive care unit
IMC: Intermediate care unit
LD: Low-density
LOS: Hospital length of stay
NGAL: Neutrophil gelatinase-associated lipocalin
PCT: Procalcitonin
PLAC8: Placenta-specific gene 8 protein
PMN: Polymorphonuclear neutrophil
qRT-PCR: Quantitative real-time polymerase chain reaction
scRNA-seq: Single-cell RNA-sequencing
SIRS: Systemic inflammatory response syndrome
SOFA: Sequential organ failure assessment
