# Supplementary material for: Transcriptional pathways of terminal differentiation in high- and low-density blood granulocytes in sepsis
Source: J Inflamm (Lond). 2024 Oct 21;21:40. doi: 10.1186/s12950-024-00414-w (PMC11492786; doi:10.1186/s12950-024-00414-w)

Additional file 1

Table of contents

		Page
**Text S1**	Clinical characteristics for the total study cohort	2
**Table S1**	GenBank Nucleotide IDs for QuantiGene^{TM} Plex Assays (Thermo Fisher Scientific) and pathway membership of genes	4
**Table S2**	Clinical characteristics for the total study cohort	11
**Table S3**	Clinical characteristics for the “granulocyte precursor cell counting” subcohort	16
**Table S4**	Clinical characteristics for the “relative granulocyte precursor abundance” subcohort	21
**Table S5**	Clinical characteristics for the “multiplex immunoassay” subcohort	26
**Table S6**	Clinical characteristics for the “QuantiGene^{TM} Plex” subcohort	31
**Table S7**	Clinical characteristics for the “qRT-PCR total granulocytes” subcohort	36
**Table S8**	Clinical characteristics for the “qRT-PCR HD & LD granulocytes” subcohort	41
**Fig. S1**	Analytical patient subcohorts	46
**Fig. S2**	Flow cytometric gating strategy to identify granulocyte precursors	47
**Fig. S3**	Radar charts comparing the analytical subcohorts to the total cohort	48
**Fig. S4**	Spearman's rank-order correlation for SOFA score values and granulocyte precursor blood counts	48
**Fig. S5**	Venn diagrams for genes with differential expression between patient subgroups across total, HD and LD granulocytes	50

Text S1

<u>Clinical characteristics for the total study cohort</u>

The SIRS etiology was 14-times abdominal surgery, four-times vascular surgery, and once each bleeding leg ulcer, craniocerebral trauma, gastrointestinal bleeding, neurosurgery, spinal surgery, and split-skin graft. In the sepsis subgroup, seven patients tested positive for Gram-negative bacteria in blood, four of which had urosepsis. One of four patients with abdominal sepsis tested positive for Gram-negative and another for both Gram-negative and -positive bacteria. One pneumonia patient tested positive for Gram-negative bacteria. The infectious focus and agent remained unclear in 13 (52%) and 15 (60%) patients, respectively, in the sepsis subgroup, and only in one (9%) and two (18%), respectively, in the septic shock subgroup. In the latter subgroup, three out of six patients with abdominal sepsis had Gram-negative, one Gram-positive, and one both classes of bacteria. In one out of two patients with a pulmonal focus (both Gram-positive) as well as in one with urosepsis (Gram-negative), the blood culture tested also positive accordingly. One additional septic shock patient had urosepsis.

The overall mean (standard deviation) age was 65.4 (12.9) years and 40% were female (Additional File 1: Table S2). General surgery was the most frequent admitting department (55%) followed by urology (23%). There were no significant subgroup differences for these features. Cardiovascular disease (43.3%), current tumor disease (40.0%), acute kidney injury (AKI) (38.3%), and diabetes (21.7%) were the most frequent concomitant diseases. Tumor disease was significantly more common in the patients with SIRS (62.5%) than sepsis (28.0%, $p = 0.0152$) and septic shock (18.2%, $p = 0.0149$). AKI rates were higher in septic shock (81.8%) than in SIRS (25.0%, $p = 0.0028$) and sepsis (32.0%, $p = 0.0058$). One quarter of the cohort was mechanically ventilated, and 65.0% received vasopressor therapy. As expected, these respective interventions were more prevalent in septic shock (81.8% and 100%) than in SIRS (8.3% and 66.7%, $p < 0.0001$ and $p = 0.0370$) and in sepsis (16.0% and 48.0%, $p = 0.0003$ and $p = 0.0026$). Values for white blood cell counts (WBCs) and C-reactive protein (CRP) were significantly higher in septic shock and sepsis than SIRS. The septic shock patients also showed higher lactate, creatinine, and international normalized ratio (INR) values and lower blood pH than the patients with SIRS and sepsis. Subgroup differences for hemoglobin, total bilirubin, and platelets as well as for body temperature, Horovitz index, and mean arterial pressure did

not reach statistical significance. The same was true for the hospital LOS, which was 24.6 (22.5) days on average.

Table S1 GenBank Nucleotide IDs for QuantiGene^{TM} Plex Assays (Thermo Fisher Scientific) and pathway memberships of genes. The name(s) of the pathway(s), which was (were) the primary reason for inclusion of a gene according to the rules described in the Results section of the main manuscript, is (are) printed in bold.

**Gene symbol**	**GenBank Nucleotide ID**[1]	**Kyoto Encyclopedia of Genes and Genomes PATHWAY**[2]	**Granule biogenesis pathway** (Grassi et al. *Cell Rep* 2018, 24(10): 2784-2794) **Endolysosomal function** (Velasquez et al., *Front Immunol* 2022, 13:864835)
ACAA2	NM_006111.2	**Fatty Acid Metabolism**	
ACADM	NM_000016.5	**Fatty Acid Metabolism**	
ACADSB	NM_001330174.1	**Fatty Acid Metabolism**	
ACAT1	NM_000019.3	**Fatty Acid Metabolism**, Carbon Metabolism	
ACLY	NM_001096.2	**TCA Cycle**	
ACO2	NM_001098.2	**TCA Cycle**, Carbon Metabolism	
ACSL5	NM_016234.3	**Fatty Acid Metabolism**	
ACSS2	NM_001076552.2	**Carbon Metabolism**	
ADGRE3	NM_032571.4		**Specific Granules**
AKIRIN1	NM_001136275	*- Reference gene -*	
ANXA1	NM_000700.2		**Secretory Vesicles**
ANXA4	NM_001153.4		**Secretory Vesicles**
ANXA5	NM_001154.3		**Secretory Vesicles**
AP3B2	NM_001278511.1	**Lysosome**	
AP4E1	NM_001252127.1	**Lysosome**	
APP[3]	NM_000484.3		**Endolysosomal Membrane**
ARSA	NM_000487.5	**Lysosome**	**Azurophilic Granules**
ATP5J2	NM_001003713.2	**OxPhos**	

Gene symbol	GenBank Nucleotide ID[1]	Kyoto Encyclopedia of Genes and Genomes PATHWAY[2]	**Granule biogenesis pathway** (Grassi et al. *Cell Rep* 2018, 24(10): 2784-2794) **Endolysosomal function** (Velasquez et al., *Front Immunol* 2022, 13:864835)
BPI	NM_001725.2		**Azurophilic Granules**
BUB1	NM_001278617.1	**Cell Cycle**	
C3AR1	NM_001326475.1		**Specific Granules**
CCNA1	NM_001111045.1	**Cell Cycle**	
CCNA2	NM_001237.4	**Cell Cycle**	
CCNB2	NM_004701.3	**Cell Cycle**	
CD14	NM_000591.3		**Secretory Vesicles**
CD177	NM_020406.3		**Tertiary Granules**
CD37	NM_001040031.1		**Cell Membrane**
CD44	NM_000610.3		**Cell Membrane**
CD53	NM_000560.3		**Cell Membrane**
CD59	NM_000611.5		**Specific Granules**
CD63	NM_001257389.1	Lysosome	**Azurophilic Granules**
CD68[3]	NM_001040059.1	**Lysosome**	**Endolysosomal Membrane**
CD82	NM_001024844.1		**Cell Membrane**
CDK1	NM_001320918.1	**Cell Cycle**	
CDK5[3]	NM_001164410.2		**Endolysosomal Cytoskeleton Adaptors**
CDKN2C	NM_001262.2	**Cell Cycle**	
CEACAM4	NM_001817.3		**Cell Membrane**
CHI3L1	NM_001276.2		**Specific Granules**
CHIT1	NM_001256125.1		**Specific Granules**
CLEC4D	NM_080387.4		**Specific Granules**
CLEC5A	NM_013252.2		**Specific Granules**

**Gene symbol**	**GenBank Nucleotide ID[1]**	**Kyoto Encyclopedia of Genes and Genomes PATHWAY[2]**	**Granule biogenesis pathway** (Grassi et al. *Cell Rep* 2018, 24(10): 2784-2794)<br>**Endolysosomal function** (Velasquez et al., *Front Immunol* 2022, 13:864835)
COX7C	NM_001867.2	**OxPhos**	
CPT1A	NM_001031847.2	**Fatty Acid Metabolism**	
CRISP3	NM_001190986.2		**Specific Granules**
CS	NM_004077.2	**TCA Cycle**,<br>Carbon Metabolism	
CST3	NM_000099.3		**Ficolin-Containing Granules**
CSTA	NM_005213.3		**Secretory Vesicles**
CTSA	NM_000308.3	**Lysosome**	**Azurophilic Granules**
CTSB	NM_001908.4	Lysosome	**Ficolin-Containing Granules**
CTSD	NM_001909.4	Lysosome	**Ficolin-containing Granules**
CTSG	NM_001911.2	**Lysosome**	Azurophilic Granules
CTSH	NM_004390.4	Lysosome	**Ficolin-containing Granules**
CTSS	NM_001199739.1	Lysosome	**Ficolin-Containing Granules**
CXCR2	NM_001168298.1		**Cell Membrane**
DIAPH2[3]	NM_006729.4		**Endolysosomal Cytoskeleton Adaptors**
E2F2	NM_004091.3	**Cell Cycle**	
FADS1	NM_013402.4	**Fatty Acid Metabolism**	
FCGR3B	NM_000570.4		**Secretory Vesicles**
FCMR	NM_001142473.1		**Cell Membrane**

**Gene symbol**	**GenBank Nucleotide ID[1]**	**Kyoto Encyclopedia of Genes and Genomes PATHWAY[2]**	**Granule biogenesis pathway** (Grassi et al. *Cell Rep* 2018, 24(10): 2784-2794) **Endolysosomal function** (Velasquez et al., *Front Immunol* 2022, 13:864835)
FIG4[3]	NM_014845.5		**Endolysosomal Cytoskeleton Adaptors**
FTH1	NM_002032.2		**Ficolin-Containing Granules**
GAPDH	NM_001256799.2	**Carbon Metabolism**	
GLB1	NM_000404.3	**Lysosome**	Azurophilic Granules
GPI	NM_000175.4	**Carbon Metabolism**	
GPR84	NM_020370.2		**Specific Granules**
GRN	NM_002087.3		**Azurophilic Granules**
GUSB	NM_000181.3	**Lysosome**	**Azurophilic Granules**
HEXA	NM_000520.5	**Lysosome**	**Azurophilic Granules**
HP	NM_001126102.2		**Specific Granules**
HSD17B12	NM_016142.2	**Fatty Acid Metabolism**	
ICAM3	NM_002162.4		**Cell Membrane**
IDH1	NM_001282386.1	**TCA Cycle**, Carbon Metabolism	
IDH3G	NM_004135.3	**TCA Cycle**, Carbon Metabolism	
IDNK	NM_001001551.3	**Carbon Metabolism**	
LAIR1	NM_001289023.2		**Specific Granules**
LAMP1	NM_005561.3	Lysosome	**Azurophilic Granules**
LCN2	NM_005564.4		**Specific Granules**
LDLR[3]	NM_000527.4		**Endolysosomal Membrane**

**Gene symbol**	**GenBank Nucleotide ID[1]**	**Kyoto Encyclopedia of Genes and Genomes PATHWAY[2]**	**Granule biogenesis pathway** (Grassi et al. *Cell Rep* 2018, 24(10): 2784-2794) **Endolysosomal function** (Velasquez et al., *Front Immunol* 2022, 13:864835)
LGALS3	NM_002306.3		**Secretory Vesicles**
LTB4R	NM_001143919.2		**Cell Membrane**
LTF	NM_001199149.1		**Specific Granules**
MAN2B1	NM_000528.3	Lysosome	**Azurophilic Granules**
MANBA	NM_005908.3	Lysosome	**Azurophilic Granules**
MCM6	NM_005915.5	**Cell Cycle**	
MDH1	NM_001199111.1	**TCA Cycle**, Carbon Metabolism	
MDH2	NM_001282403.1	**TCA Cycle**, Carbon Metabolism	
MMP8	NM_001304441.1		**Specific Granules**
MMP9	NM_004994.2		**Tertiary Granules**
MPO	NM_000250.1		**Azurophilic Granules**
MRPL18	NM_014161.4	**Ribosome**	
MS4A3	NM_001031666.1	**Secondary granules**	
NDUFA4	NM_002489.3	**OxPhos**	
NDUFA9	NM_005002.4	**OxPhos**	
NDUFB5	NM_001199957.1	**OxPhos**	
NDUFB9	NP_001298097.1	**OxPhos**	
NDUFS3	NM_004551.2	**OxPhos**	
NPC1	NM_000271.4	**Lysosome**	
OLFM4	NM_006418.4		**Specific Granules**
PDHA1	NM_000284.3	**TCA Cycle, Carbon Metabolism**	
PECR	NM_018441.5	**Fatty Acid Metabolism**	

**Gene symbol**	**GenBank Nucleotide ID**[1]	**Kyoto Encyclopedia of Genes and Genomes PATHWAY**[2]	**Granule biogenesis pathway** (Grassi et al. *Cell Rep* 2018, 24(10): 2784-2794) **Endolysosomal function** (Velasquez et al., *Front Immunol* 2022, 13:864835)
PKM	NM_001206796.2	**Carbon Metabolism**	
PLA2G7	NM_001168357.1		**Cell Membrane**
PLAC8/LGALS13	NM_013268.2		**Azurophilic Granules**
PLD1	NM_002662.4		**Specific Granules**
PPA2	NM_006903.4	**OxPhos**	
PRTN3	NM_002777.3		**Azurophilic Granules**
PSMA1	NM_002786.3	**Proteasome**	
PSMA5	NM_001199772.1	**Proteasome**	
PSMA6	NM_001282232.1	**Proteasome**	
PSMB2	NM_002794.4	**Proteasome**	
PSMB6	NM_002798.2	**Proteasome**	
PSMC2	NM_001204453.1	**Proteasome**	
PSMD1	NM_001191037.1	**Proteasome**	
PSMD11	NM_001270482.1	**Proteasome**	
PSMD14	NM_005805.5	**Proteasome**	
PSMD3	NM_002809.3	**Proteasome**	
PTX3	NM_002852.3		**Specific Granules**
RBX1	NM_014248.3	**Cell Cycle**	
RNASE2	NM_002934.2		**Azurophilic Granules**
RNASE3	NM_002935.2		**Azurophilic Granules**
RPL18	NM_000979.3	**Ribosome**	
RPL24	NM_000986.3	**Ribosome**	
RPL27	NM_000988.3	**Ribosome**	

**Gene symbol**	**GenBank Nucleotide ID**[1]	**Kyoto Encyclopedia of Genes and Genomes PATHWAY**[2]	**Granule biogenesis pathway** (Grassi et al. *Cell Rep* 2018, 24(10): 2784-2794) **Endolysosomal function** (Velasquez et al., *Front Immunol* 2022, 13:864835)
RPL35	NM_007209.3	**Ribosome**	
RPL4	NM_000968.3	**Ribosome**	
RPL5	NM_000969.3	**Ribosome**	
RPS15	NM_001018.4	**Ribosome**	
RPS15A	NM_001019.4	**Ribosome**	
RPS27L	NM_015920.3	**Ribosome**	
SDHC	NM_001035511.1	**TCA Cycle**, OxPhos	
SGSH[3]	NM_000199.3	**Lysosome**	
SUCLA2	NM_003850.2	**TCA Cycle**, Carbon Metabolism	
TCN1	NM_001062.3		**Specific Granules**
TECR	NM_001321170.1	**Fatty Acid Metabolism**	
TPP1[3]	NM_000391.3	**Lysosome**	**Endolysosomal Hydrolase**

[1] https://www.ncbi.nlm.nih.gov/genbank/

[2] Only the canonical pathways of early terminal granulocytic differentiation according to Theilgaard-Mönch et al. (2005) are identified (Theilgaard-Mönch et al. *Blood* 2005, 105(4):1785-1796): Carbon Metabolism, Cell Cycle, Fatty Acid Metabolism, Lysosome, OxPhos, Proteasome, Ribosome, TCA Cycle.

[3] Genes included because differential expression in sepsis and SIRS on intensive care unit admission was previously validated in our lab (Velasquez et al., *Front Immunol* 2022, 13:864835).

Table S2 **Clinical characteristics for the total study cohort**

	Total (N=60)		
	Median (IQR) n (%)	Mean (SD)	Missing n (%)
**Demographic data**			
Age (years)	67 (59.5–72)	65.4 (12.9)	
Male	36 (60.0%)		
**Admitting department**			
Anesthesiology	1 (1.67%)		
Dermatology	1 (1.67%)		
General surgery	33 (55.0%)		
Gynecology	1 (1.67%)		
Nephrology	1 (1.67%)		
Neurosurgery	4 (6.67%)		
Orthopedics and trauma center	5 (8.33%)		
Urology	14 (23.3%)		
**Concomitant diseases**			
Diabetes	13 (21.7%)		
Cardiovascular disease	26 (43.3%)		
Respiratory disease	7 (11.7%)		
Alcohol abuse	1 (1.67%)		
Acute kidney injury	23 (38.3%)		
Current tumor disease	24 (40.0%)		
**Hospital mortality**	16 (26.7%)		
**Hospital length of stay (days)**	17.8 (11.2–33.6)	24.6 (22.5)	
**SOFA score**	4.50 (3–7)	5.48 (3.47)	
**Mechanical ventilation**	15 (25.0%)		
**Vasopressor therapy**	39 (65.0%)		

Table S2 **Clinical characteristics for the total study cohort**

	Total (N=60)		
	Median (IQR) n (%)	Mean (SD)	Missing n (%)
**Lab parameters**			
White blood cell count (10^9/l)	15.5 (9.87–19.8)	17.0 (10.7)	
CRP (mg/l)	150 (81–256)	168 (111)	
Lactate (mmol/l)	1.40 (0.90–2.20)	2.72 (5.06)	
pH	7.41 (7.38–7.44)	7.40 (0.06)	
Hemoglobin (g/dl)	9.10 (8.05–10.4)	9.48 (1.94)	
Creatinine (mg/dl)	1.17 (0.83–2)	1.65 (1.23)	2 (3.33%)
Total bilirubin (µmol/l)	14.2 (7.27–22.8)	19.4 (20.2)	
Platelets (10^9/l)	207 (152–308)	242 (134)	
International Normalized Ratio (INR)	1.13 (1.09–1.25)	1.28 (0.46)	
**Vital signs**			
Temperature (°C)	36.6 (36.1–37.0)	36.6 (0.86)	
Horovitz Index (mmHG)	376 (240–471)	373 (154)	3 (5.00%)
Mean arterial pressure (mmHG)	81 (70–90)	80.4 (14.0)	

Table S2 **Clinical characteristics for the total study cohort**

	SIRS (N=24)			Sepsis (N=25)			Septic shock (N=11)	
	Median (IQR) n (%)	Mean (SD)	Missing n (%)	Median (IQR) n (%)	Mean (SD)	Missing n (%)	Median (IQR) n (%)	Mean (SD)
**Demographic data**								
Age (years)	67.5 (58.5–72)	63.8 (12.8)		66 (56–74)	64.9 (13.3)		68 (64–81)	70.3 (12.0)
Male	14 (58.3%)			15 (60.0%)			7 (63.6%)	
**Admitting department**								
Anesthesiology				1 (4.00%)				
Dermatology	1 (4.17%)							
General surgery	11 (45.8%)			15 (60.0%)			7 (63.6%)	
Gynecology	1 (4.17%)							
Nephrology							1 (9.09%)	
Neurosurgery	3 (12.5%)						1 (9.09%)	
Orthopedics and trauma center	1 (4.17%)			3 (12.0%)			1 (9.09%)	
Urology	7 (29.2%)			6 (24.0%)			1 (9.09%)	
**Concomitant diseases**								
Diabetes	5 (20.8%)			2 (8.00%)			6 (54.5%)	
Cardiovascular disease	11 (45.8%)			9 (36.0%)			6 (54.5%)	
Respiratory disease	2 (8.33%)			3 (12.0%)			2 (18.2%)	
Alcohol abuse	1 (4.17%)			0 (0%)			0 (0%)	
Acute kidney injury	6 (25.0%)			8 (32.0%)			9 (81.8%)	
Current tumor disease	15 (62.5%)			7 (28.0%)			2 (18.2%)	
**Hospital mortality**	3 (12.5%)			4 (16.0%)			9 (81.8%)	
**Hospital length of stay (days)**	17.5 (11.7–36.2)	23.8 (16.4)		16.2 (11.2–22.2)	21.2 (16.2)		21.2 (5.55–46.3)	34.0 (40.2)
**SOFA score**	4 (2–5)	4.04 (1.88)		4 (3–6)	4.48 (2.33)		10 (9–14)	10.9 (3.18)
**Mechanical ventilation**	2 (8.33%)			4 (16.0%)			9 (81.8%)	

Table S2 Clinical characteristics for the total study cohort

	SIRS (N=24)			Sepsis (N=25)			Septic shock (N=11)	
	Median (IQR) n (%)	Mean (SD)	Missing n (%)	Median (IQR) n (%)	Mean (SD)	Missing n (%)	Median (IQR) n (%)	Mean (SD)
**Vasopressor therapy**	16 (66.7%)			12 (48.0%)			11 (100%)	
**Lab parameters**								
White blood cell count (10^9/l)	12.3 (8.97–15.9)	12.6 (3.36)		17.2 (9.40–24.5)	18.0 (9.86)		19.8 (12.9–24.0)	24.5 (17.5)
CRP (mg/l)	93 (48–135)	96.8 (62.5)		214 (137–282)	211 (99.8)		239 (128–295)	227 (137)
Lactate (mmol/l)	1.35 (0.90–2)	1.58 (0.98)		1.20 (0.80–1.90)	1.36 (0.74)		3.10 (1.50–19)	8.32 (10.3)
pH	7.44 (7.40–7.46)	7.43 (0.03)		7.41 (7.39–7.43)	7.41 (0.04)		7.33 (7.29–7.37)	7.33 (0.06)
Hemoglobin (g/dl)	9 (8.35–10.3)	9.40 (1.42)		9 (7.70–10.4)	9.52 (2.50)		9.20 (8.50–10.9)	9.55 (1.56)
Creatinine (mg/dl)	0.95 (0.71–1.17)	1.34 (1.27)		1.30 (0.81–2)	1.56 (1.09)	2 (8.00%)	2.30 (1.43–3.38)	2.50 (1.13)
Total bilirubin (µmol/l)	13.3 (6.41–18.8)	17.2 (22.1)		14.0 (8.89–25.3)	18.3 (15.5)		19.2 (10.3–30.6)	26.7 (25.1)
Platelets (10^9/l)	243 (165–301)	248 (90.3)		209 (140–313)	253 (149)		137 (72–307)	201 (177)
International Normalized Ratio (INR)	1.11 (1.07–1.15)	1.14 (0.17)		1.13 (1.09–1.25)	1.28 (0.39)		1.30 (1.20–1.72)	1.60 (0.81)
**Vital signs**								
Temperature (°C)	36.5 (35.9–36.8)	36.3 (0.79)		36.7 (36.3–36.9)	36.6 (0.68)		36.5 (35.9–38.2)	36.9 (1.24)
Horovitz Index (mmHG)	371 (338–519)	406 (155)	1 (4.17%)	381 (178–514)	361 (156)	2 (8.00%)	287 (215–453)	332 (145)
Mean arterial pressure (mmHG)	80 (70.5–97)	82.3 (15.4)		83 (78–86)	82.1 (10.7)		73 (64–86)	72.3 (15.6)

Table S2 **Clinical characteristics for the total study cohort**

	Total (norm.)	SIRS vs Sepsis	SIRS vs Septic shock	Sepsis vs Septic shock
	p	p	p	p
Age (years)	0.2538	0.7632	0.1583	0.2421
Male		0.9055	1.0000~	1.0000~
Admitting department		0.2623~	0.5392~	0.3518~
Diabetes		0.2467~	0.0623~	0.0049~
Cardiovascular disease		0.4839	0.6321	0.4646~
Respiratory disease		1.0000~	0.5748~	0.6309~
Alcohol abuse		0.4898~	1.0000~	
Acute kidney injury		0.5877	0.0028~	0.0058
Current tumor disease		0.0152	0.0149	0.6896~
Hospital mortality		1.0000~	0.0001~	0.0003~
Hospital length of stay (days)	<.0001	0.5863	0.4354	0.3317
SOFA score	<.0001	0.4711	<.0001	<.0001
Mechanical ventilation		0.6671~	<.0001~	0.0003~
Vasopressor therapy		0.1869	0.0370~	0.0026~
White blood cell count (10^9/l)	<.0001	0.0154	0.0488	0.2694
CRP (mg/l)	0.0364	<.0001	0.0109	0.7389
Lactate (mmol/l)	<.0001	0.4020	0.0546	0.0485
pH	0.0290	0.0511	<.0001	0.0006
Hemoglobin (g/dl)	0.0048	0.8312	0.7899	0.9707
Creatinine (mg/dl)	<.0001	0.5283	0.0128	0.0331
Total bilirubin (µmol/l)	<.0001	0.8496	0.2987	0.3227
Platelets (10^9/l)	0.0029	0.8784	0.4220	0.4047
International Normalized Ratio (INR)	<.0001	0.0905	0.0867	0.2381
Temperature (°C)	0.3692	0.1736	0.1508	0.4253
Horovitz Index (mmHG)	0.0797	0.3303	0.1918	0.6103
Mean arterial pressure (mmHG)	0.9014	0.9556	0.0921	0.0776

p-value: t-test (method Satterthwaite) for continuous parameters, #: Mann-Whitney-Wilcoxon test (U test); Chi² test for categorical parameters, ~: Fisher's exact test

Table S3 Clinical characteristics for the "granulocyte precursor cell counting" subcohort

	Total (N=51)		
	Median (IQR) n (%)	Mean (SD)	Missing n (%)
**Demographic data**			
Age (years)	68 (59–74)	65.8 (13.4)	
Male	30 (58.8%)		
**Admitting department**			
Anesthesiology	1 (1.96%)		
Dermatology	1 (1.96%)		
General surgery	28 (54.9%)		
Nephrology	1 (1.96%)		
Neurosurgery	3 (5.88%)		
Orthopedics and trauma center	5 (9.80%)		
Urology	12 (23.5%)		
**Concomitant diseases**			
Diabetes	13 (25.5%)		
Cardiovascular disease	22 (43.1%)		
Respiratory disease	6 (11.8%)		
Alcohol abuse	1 (1.96%)		
Acute kidney injury	21 (41.2%)		
Current tumor disease	20 (39.2%)		
**Hospital mortality**	14 (27.5%)		
**Hospital length of stay (days)**	17.3 (10.8–33.0)	24.3 (23.4)	
**SOFA score**	4 (3–7)	5.69 (3.58)	
**Mechanical ventilation**	13 (25.5%)		
**Vasopressor therapy**	33 (64.7%)		

Table S3 **Clinical characteristics for the "granulocyte precursor cell counting" subcohort**

	Total (N=51)		
	Median (IQR) n (%)	Mean (SD)	Missing n (%)
**Lab parameters**			
White blood cell count (10^9/l)	15.9 (9.65–19.8)	17.2 (11.1)	
CRP (mg/l)	148 (76–259)	166 (113)	
Lactate (mmol/l)	1.50 (0.90–2.30)	3 (5.45)	
pH	7.40 (7.36–7.44)	7.40 (0.06)	
Hemoglobin (g/dl)	9.20 (8.20–10.7)	9.61 (1.96)	
Creatinine (mg/dl)	1.14 (0.86–2.08)	1.72 (1.30)	2 (3.92%)
Total bilirubin (µmol/l)	14.9 (6.84–23.9)	19.8 (21.3)	
Platelets (10^9/l)	203 (151–307)	239 (134)	
International Normalized Ratio (INR)	1.13 (1.09–1.26)	1.31 (0.49)	
**Vital signs**			
Temperature (°C)	36.6 (36.1–37.1)	36.6 (0.85)	
Horovitz Index (mmHG)	381 (225–471)	364 (153)	2 (3.92%)
Mean arterial pressure (mmHG)	81 (69–91)	79.6 (13.9)	

Table S3 Clinical characteristics for the “granulocyte precursor cell counting” subcohort

	SIRS (N=18)		Sepsis (N=22)			Septic shock (N=11)	
	Median (IQR) n (%)	Mean (SD)	Median (IQR) n (%)	Mean (SD)	Missing n (%)	Median (IQR) n (%)	Mean (SD)
**Demographic data**							
Age (years)	68 (61–72)	64.8 (13.6)	63 (53–76)	64.4 (14.0)		68 (64–81)	70.3 (12.0)
Male	11 (61.1%)		12 (54.5%)			7 (63.6%)	
**Admitting department**							
Anesthesiology			1 (4.55%)				
Dermatology	1 (5.56%)						
General surgery	8 (44.4%)		13 (59.1%)			7 (63.6%)	
Nephrology						1 (9.09%)	
Neurosurgery	2 (11.1%)					1 (9.09%)	
Orthopedics and trauma center	1 (5.56%)		3 (13.6%)			1 (9.09%)	
Urology	6 (33.3%)		5 (22.7%)			1 (9.09%)	
**Concomitant diseases**							
Diabetes	5 (27.8%)		2 (9.09%)			6 (54.5%)	
Cardiovascular disease	8 (44.4%)		8 (36.4%)			6 (54.5%)	
Respiratory disease	1 (5.56%)		3 (13.6%)			2 (18.2%)	
Alcohol abuse	1 (5.56%)		0 (0%)			0 (0%)	
Acute kidney injury	5 (27.8%)		7 (31.8%)			9 (81.8%)	
Current tumor disease	11 (61.1%)		7 (31.8%)			2 (18.2%)	
**Hospital mortality**	2 (11.1%)		3 (13.6%)			9 (81.8%)	
**Hospital length of stay (days)**	17.5 (11.2–31.6)	21.6 (14.8)	15.4 (10.8–23.3)	21.7 (17.2)		21.2 (5.55–46.3)	34.0 (40.2)
**SOFA score**	4 (3–6)	4.44 (1.79)	3.50 (3–5)	4.09 (2.20)		10 (9–14)	10.9 (3.18)
**Mechanical ventilation**	2 (11.1%)		2 (9.09%)			9 (81.8%)	
**Vasopressor therapy**	13 (72.2%)		9 (40.9%)			11 (100%)	
**Lab parameters**							

Table S3 Clinical characteristics for the "granulocyte precursor cell counting" subcohort

	SIRS (N=18)		Sepsis (N=22)			Septic shock (N=11)	
	Median (IQR) n (%)	Mean (SD)	Median (IQR) n (%)	Mean (SD)	Missing n (%)	Median (IQR) n (%)	Mean (SD)
White blood cell count (10^9/l)	14.3 (9.10–16.0)	13.2 (3.55)	15.0 (9–23.4)	16.9 (9.93)		19.8 (12.9–24.0)	24.5 (17.5)
CRP (mg/l)	81 (37–106)	87.1 (54.8)	207 (125–282)	201 (101)		239 (128–295)	227 (137)
Lactate (mmol/l)	1.45 (0.90–2.20)	1.73 (1.09)	1.25 (0.80–1.90)	1.38 (0.77)		3.10 (1.50–19)	8.32 (10.3)
pH	7.44 (7.40–7.46)	7.43 (0.04)	7.41 (7.39–7.43)	7.41 (0.05)		7.33 (7.29–7.37)	7.33 (0.06)
Hemoglobin (g/dl)	9.35 (8.40–10.3)	9.49 (1.32)	9.15 (7.90–10.8)	9.73 (2.56)		9.20 (8.50–10.9)	9.55 (1.56)
Creatinine (mg/dl)	0.97 (0.73–1.14)	1.42 (1.40)	1.13 (0.79–1.89)	1.55 (1.17)	2 (9.09%)	2.30 (1.43–3.38)	2.50 (1.13)
Total bilirubin (µmol/l)	13.9 (6.33–19.0)	18.1 (25.3)	14.1 (8.89–25.3)	17.7 (15.0)		19.2 (10.3–30.6)	26.7 (25.1)
Platelets (10^9/l)	221 (157–292)	233 (86.9)	212 (174–313)	262 (142)		137 (72–307)	201 (177)
International Normalized Ratio (INR)	1.10 (1.05–1.15)	1.14 (0.19)	1.13 (1.09–1.25)	1.30 (0.41)		1.30 (1.20–1.72)	1.60 (0.81)
**Vital signs**							
Temperature (°C)	36.3 (36–37)	36.4 (0.68)	36.7 (36.2–36.9)	36.6 (0.71)		36.5 (35.9–38.2)	36.9 (1.24)
Horovitz Index (mmHG)	395 (343–519)	406 (150)	379 (170–493)	344 (158)	2 (9.09%)	287 (215–453)	332 (145)
Mean arterial pressure (mmHG)	75 (69–99)	81.5 (16.6)	83.5 (78–86)	81.7 (9.00)		73 (64–86)	72.3 (15.6)

Table S3 Clinical characteristics for the "granulocyte precursor cell counting" subcohort

	Total (norm.)	SIRS vs Sepsis	SIRS vs Septic shock	Sepsis vs Septic shock
	p	p	p	p
Age (years)	0.2785	0.9254	0.2669	0.2201
Male		0.6760	1.0000~	0.7193~
Admitting department		0.3146~	0.4653~	0.4448~
Diabetes		0.2110~	0.2399~	0.0082~
Cardiovascular disease		0.6038	0.5974	0.4587~
Respiratory disease		0.6133~	0.5394~	1.0000~
Alcohol abuse		0.4500~	1.0000~	
Acute kidney injury		0.7815	0.0047	0.0067
Current tumor disease		0.0639	0.0524~	0.6808~
Hospital mortality		1.0000~	0.0002~	0.0002~
Hospital length of stay (days)	<.0001	0.9934	0.3480	0.3511
SOFA score	<.0001	0.5786	<.0001	<.0001
Mechanical ventilation		1.0000~	0.0002~	<.0001~
Vasopressor therapy		0.0477	0.1261~	0.0016~
White blood cell count (10^9/l)	<.0001	0.1092	0.0584	0.2058
CRP (mg/l)	0.0297	<.0001	0.0072	0.5923
Lactate (mmol/l)	<.0001	0.2502	0.0596	0.0489
pH	0.1088	0.0830	<.0001	0.0006
Hemoglobin (g/dl)	0.0149	0.7128	0.9289	0.8025
Creatinine (mg/dl)	<.0001	0.7677	0.0321	0.0379
Total bilirubin (µmol/l)	<.0001	0.9507	0.3866	0.2956
Platelets (10^9/l)	0.0008	0.4435	0.5826	0.3377
International Normalized Ratio (INR)	<.0001	0.1293	0.0926	0.2620
Temperature (°C)	0.4302	0.4537	0.2143	0.3836
Horovitz Index (mmHG)	0.0367	0.2270	0.2045	0.8324
Mean arterial pressure (mmHG)	0.9336	0.9588	0.1463	0.0855

p-value: t-test (method Satterthwaite) for continuous parameters, #: Mann-Whitney-Wilcoxon test (U test); Chi² test for categorical parameters, ~: Fisher's exact test

Table S4 **Clinical characteristics for the “relative granulocyte precursor abundance” subcohort**

	**Total (N=42)**		
	**Median (IQR) n (%)**	**Mean (SD)**	**Missing n (%)**
**Demographic data**			
Age (years)	68.5 (60–77)	67.0 (14.0)	
Male	24 (57.1%)		
**Admitting department**			
Anesthesiology	1 (2.38%)		
Dermatology	1 (2.38%)		
General surgery	23 (54.8%)		
Gynecology	1 (2.38%)		
Neurosurgery	3 (7.14%)		
Orthopedics and trauma center	3 (7.14%)		
Urology	10 (23.8%)		
**Concomitant diseases**			
Diabetes	9 (21.4%)		
Cardiovascular disease	19 (45.2%)		
Respiratory disease	3 (7.14%)		
Alcohol abuse	1 (2.38%)		
Acute kidney injury	14 (33.3%)		
Current tumor disease	18 (42.9%)		
**Hospital mortality**	10 (23.8%)		
**Hospital length of stay (days)**	18.9 (10.3–33.0)	22.6 (15.7)	
**SOFA score**	4 (3–7)	5.45 (3.68)	
**Mechanical ventilation**	10 (23.8%)		
**Vasopressor therapy**	26 (61.9%)		

Table S4 **Clinical characteristics for the "relative granulocyte precursor abundance" subcohort**

	**Total (N=42)**		
	**Median (IQR) n (%)**	**Mean (SD)**	**Missing n (%)**
**Lab parameters**			
White blood cell count (10^9/l)	15.5 (9.65–17.8)	17.4 (11.9)	
CRP (mg/l)	155 (76–240)	164 (104)	
Lactate (mmol/l)	1.45 (0.90–2.20)	2.00 (2.88)	
pH	7.40 (7.38–7.44)	7.40 (0.05)	
Hemoglobin (g/dl)	9.25 (8.40–10.4)	9.63 (1.78)	
Creatinine (mg/dl)	1.07 (0.85–2.02)	1.63 (1.24)	2 (4.76%)
Total bilirubin (µmol/l)	15.3 (8.89–23.9)	21.1 (23.0)	
Platelets (10^9/l)	206 (153–292)	243 (140)	
International Normalized Ratio (INR)	1.13 (1.10–1.25)	1.25 (0.29)	
**Vital signs**			
Temperature (°C)	36.6 (36.1–37.1)	36.6 (0.94)	
Horovitz Index (mmHG)	395 (257–518)	394 (160)	2 (4.76%)
Mean arterial pressure (mmHG)	81 (70–86)	79.9 (13.2)	

Table S4 **Clinical characteristics for the "relative granulocyte precursor abundance" subcohort**

	SIRS (N=18)		Sepsis (N=17)			Septic shock (N=7)	
	Median (IQR) n (%)	Mean (SD)	Median (IQR) n (%)	Mean (SD)	Missing n (%)	Median (IQR) n (%)	Mean (SD)
**Demographic data**							
Age (years)	69 (61–72)	65.1 (13.7)	68 (59–79)	66.5 (14.4)		69 (67–87)	73.1 (14.4)
Male	10 (55.6%)		9 (52.9%)			5 (71.4%)	
**Admitting department**							
Anesthesiology			1 (5.88%)				
Dermatology	1 (5.56%)						
General surgery	9 (50.0%)		9 (52.9%)			5 (71.4%)	
Gynecology	1 (5.56%)						
Neurosurgery	2 (11.1%)					1 (14.3%)	
Orthopedics and trauma center	1 (5.56%)		2 (11.8%)				
Urology	4 (22.2%)		5 (29.4%)			1 (14.3%)	
**Concomitant diseases**							
Diabetes	5 (27.8%)		1 (5.88%)			3 (42.9%)	
Cardiovascular disease	8 (44.4%)		7 (41.2%)			4 (57.1%)	
Respiratory disease	1 (5.56%)		2 (11.8%)			0 (0%)	
Alcohol abuse	1 (5.56%)		0 (0%)			0 (0%)	
Acute kidney injury	4 (22.2%)		4 (23.5%)			6 (85.7%)	
Current tumor disease	11 (61.1%)		6 (35.3%)			1 (14.3%)	
**Hospital mortality**	2 (11.1%)		2 (11.8%)			6 (85.7%)	
**Hospital length of stay (days)**	20.8 (11.2–38.4)	25.4 (17.0)	16.4 (10.3–22.2)	20.5 (14.9)		18.2 (5.55–32.7)	20.3 (15.4)
**SOFA score**	4 (3–6)	4.33 (1.88)	3 (2–4)	3.94 (2.44)		13 (9–14)	12 (2.58)
**Mechanical ventilation**	2 (11.1%)		2 (11.8%)			6 (85.7%)	
**Vasopressor therapy**	12 (66.7%)		7 (41.2%)			7 (100%)	

Table S4 **Clinical characteristics for the "relative granulocyte precursor abundance" subcohort**

	SIRS (N=18)		Sepsis (N=17)			Septic shock (N=7)	
	Median (IQR) n (%)	Mean (SD)	Median (IQR) n (%)	Mean (SD)	Missing n (%)	Median (IQR) n (%)	Mean (SD)
**Lab parameters**							
White blood cell count (10^9/l)	13.1 (9.10–15.9)	12.9 (3.40)	16.5 (9.40–24.5)	18.0 (10.2)		20.2 (10.1–58.1)	27.7 (21.5)
CRP (mg/l)	81 (37–162)	99.6 (69.0)	214 (137–281)	215 (93.0)		239 (47–295)	205 (126)
Lactate (mmol/l)	1.35 (0.80–2.20)	1.49 (0.86)	1.20 (0.80–1.70)	1.28 (0.69)		2.10 (1.50–5.50)	5.09 (6.32)
pH	7.43 (7.40–7.46)	7.43 (0.04)	7.42 (7.39–7.43)	7.41 (0.04)		7.33 (7.29–7.37)	7.33 (0.04)
Hemoglobin (g/dl)	9 (8.30–10.3)	9.36 (1.39)	9.30 (8–10.4)	9.76 (2.26)		9.40 (9.10–11.5)	10 (1.51)
Creatinine (mg/dl)	0.95 (0.73–1.19)	1.50 (1.42)	1.04 (0.81–1.50)	1.39 (0.94)	2 (11.8%)	2.85 (1.41–3.38)	2.45 (1.09)
Total bilirubin (µmol/l)	16.3 (6.33–19.8)	19.4 (25.3)	14.0 (9.41–20.7)	19.0 (17.3)		21.7 (14.2–30.6)	30.6 (29.5)
Platelets (10^9/l)	224 (157–292)	237 (90.6)	209 (174–292)	261 (154)		137 (57–307)	216 (215)
International Normalized Ratio (INR)	1.12 (1.09–1.15)	1.15 (0.19)	1.16 (1.09–1.25)	1.28 (0.35)		1.41 (1.22–1.72)	1.44 (0.27)
**Vital signs**							
Temperature (°C)	36.3 (35.8–36.8)	36.2 (0.83)	36.7 (36.4–37.1)	36.7 (0.74)		36.7 (36.4–38.6)	37.3 (1.30)
Horovitz Index (mmHG)	395 (338–533)	423 (171)	381 (225–518)	371 (150)	2 (11.8%)	410 (215–483)	367 (160)
Mean arterial pressure (mmHG)	80.5 (69–99)	82.1 (16.3)	83 (78–84)	80.9 (8.67)		73 (64–80)	72.1 (12.3)

Table S4 **Clinical characteristics for the “relative granulocyte precursor abundance” subcohort**

	Total (norm.)	SIRS vs Sepsis	SIRS vs Septic shock	Sepsis vs Septic shock
	p	p	p	p
Age (years)	0.0864	0.7675	0.2307	0.3270
Male		0.8767	0.6592~	0.6529~
Admitting department		0.6974~	1.0000~	0.5183~
Diabetes		0.1774~	0.6396~	0.0593~
Cardiovascular disease		0.8452	0.6728~	0.6591~
Respiratory disease		0.6026~	1.0000~	1.0000~
Alcohol abuse		1.0000~	1.0000~	
Acute kidney injury		1.0000~	0.0068~	0.0088~
Current tumor disease		0.1267	0.0730~	0.6245~
Hospital mortality		1.0000~	0.0010~	0.0013~
Hospital length of stay (days)	0.0021	0.3755	0.4903	0.9781
SOFA score	<.0001	0.5992	<.0001	<.0001
Mechanical ventilation		1.0000~	0.0010~	0.0013~
Vasopressor therapy		0.1303	0.1372~	0.0188~
White blood cell count (10^9/l)	<.0001	0.0672	0.1191	0.2887
CRP (mg/l)	0.1489	0.0002	0.0731	0.8479
Lactate (mmol/l)	<.0001	0.4373	0.1834	0.1627
pH	0.2772	0.1730	0.0005	0.0018
Hemoglobin (g/dl)	0.0563	0.5325	0.3508	0.7645
Creatinine (mg/dl)	<.0001	0.7972	0.0942	0.0503
Total bilirubin (µmol/l)	<.0001	0.9555	0.3975	0.3596
Platelets (10^9/l)	0.0009	0.5814	0.8148	0.6310
International Normalized Ratio (INR)	<.0001	0.2082	0.0318	0.2352
Temperature (°C)	0.5593	0.0858	0.0857	0.3138
Horovitz Index (mmHG)	0.2513	0.3568	0.4584	0.9634
Mean arterial pressure (mmHG)	0.8283	0.7905	0.1207	0.1218

p-value: t-test (method Satterthwaite) for continuous parameters, #: Mann-Whitney-Wilcoxon test (U test); Chi² test for categorical parameters, ~: Fisher's exact test

Table S5 **Clinical characteristics for the "multiplex immunoassay" subcohort**

	Total (N=53)		
	**Median (IQR) n (%)**	**Mean (SD)**	**Missing n (%)**
**Demographic data**			
Age (years)	68 (59–72)	65.8 (13.2)	
Male	32 (60.4%)		
**Admitting department**			
Anesthesiology	1 (1.89%)		
Dermatology	1 (1.89%)		
General surgery	28 (52.8%)		
Gynecology	1 (1.89%)		
Neurosurgery	3 (5.66%)		
Orthopedics and trauma center	5 (9.43%)		
Urology	14 (26.4%)		
**Concomitant diseases**			
Diabetes	12 (22.6%)		
Cardiovascular disease	21 (39.6%)		
Respiratory disease	5 (9.43%)		
Alcohol abuse	1 (1.89%)		
Acute kidney injury	20 (37.7%)		
Current tumor disease	21 (39.6%)		
**Hospital mortality**	15 (28.3%)		
**Hospital length of stay (days)**	18.9 (11.2–33.0)	23.2 (16.2)	
**SOFA score**	4 (3–7)	5.53 (3.58)	
**Mechanical ventilation**	13 (24.5%)		
**Vasopressor therapy**	34 (64.2%)		
**Lab parameters**			

Table S5 **Clinical characteristics for the “multiplex immunoassay” subcohort**

	Total (N=53)		
	Median (IQR) n (%)	Mean (SD)	Missing n (%)
White blood cell count (10^9/l)	15.6 (10.1–19.8)	17.1 (11.0)	
CRP (mg/l)	148 (79–252)	166 (105)	
Lactate (mmol/l)	1.50 (0.90–2.20)	2.87 (5.36)	
pH	7.40 (7.37–7.44)	7.40 (0.06)	
Hemoglobin (g/dl)	9.20 (8.30–10.4)	9.53 (1.96)	
Creatinine (mg/dl)	1.14 (0.83–2.08)	1.67 (1.29)	2 (3.77%)
Total bilirubin (µmol/l)	15.2 (7.70–23.9)	20.1 (21.2)	
Platelets (10^9/l)	206 (151–299)	235 (136)	
International Normalized Ratio (INR)	1.13 (1.09–1.25)	1.30 (0.48)	
**Vital signs**			
Temperature (°C)	36.6 (36.1–37)	36.5 (0.89)	
Horovitz Index (mmHG)	381 (230–514)	379 (158)	2 (3.77%)
Mean arterial pressure (mmHG)	80 (70–86)	79.7 (12.6)	

Table S5 **Clinical characteristics for the “multiplex immunoassay” subcohort**

	SIRS (N=21)		Sepsis (N=23)			Septic shock (N=9)	
	Median (IQR) n (%)	Mean (SD)	Median (IQR) n (%)	Mean (SD)	Missing n (%)	Median (IQR) n (%)	Mean (SD)
**Demographic data**							
Age (years)	68 (60–72)	64.5 (12.8)	66 (53–76)	64.6 (13.7)		69 (67–81)	72 (12.6)
Male	12 (57.1%)		14 (60.9%)			6 (66.7%)	
**Admitting department**							
Anesthesiology			1 (4.35%)				
Dermatology	1 (4.76%)						
General surgery	9 (42.9%)		13 (56.5%)			6 (66.7%)	
Gynecology	1 (4.76%)						
Neurosurgery	2 (9.52%)					1 (11.1%)	
Orthopedics and trauma center	1 (4.76%)		3 (13.0%)			1 (11.1%)	
Urology	7 (33.3%)		6 (26.1%)			1 (11.1%)	
**Concomitant diseases**							
Diabetes	5 (23.8%)		2 (8.70%)			5 (55.6%)	
Cardiovascular disease	8 (38.1%)		9 (39.1%)			4 (44.4%)	
Respiratory disease	1 (4.76%)		3 (13.0%)			1 (11.1%)	
Alcohol abuse	1 (4.76%)		0 (0%)			0 (0%)	
Acute kidney injury	5 (23.8%)		7 (30.4%)			8 (88.9%)	
Current tumor disease	14 (66.7%)		6 (26.1%)			1 (11.1%)	
**Hospital mortality**	3 (14.3%)		4 (17.4%)			8 (88.9%)	
**Hospital length of stay (days)**	19.0 (12.1–34.1)	23.8 (16.2)	16.4 (10.8–23.3)	22.0 (16.7)		21.2 (17.3–35.8)	25.1 (16.6)
**SOFA score**	4 (3–5)	4.24 (1.81)	4 (3–5)	4.22 (2.24)		13 (9–14)	11.9 (2.57)
**Mechanical ventilation**	2 (9.52%)		3 (13.0%)			8 (88.9%)	
**Vasopressor therapy**	14 (66.7%)		11 (47.8%)			9 (100%)	
**Lab parameters**							

Table S5 Clinical characteristics for the "multiplex immunoassay" subcohort

	SIRS (N=21)		Sepsis (N=23)			Septic shock (N=9)	
	Median (IQR) n (%)	Mean (SD)	Median (IQR) n (%)	Mean (SD)	Missing n (%)	Median (IQR) n (%)	Mean (SD)
White blood cell count (10^9/l)	13.0 (11.8–15.9)	13.1 (3.28)	17.2 (9–24.5)	17.7 (9.85)		19.8 (12.9–20.8)	25.2 (19.4)
CRP (mg/l)	95 (59–148)	101 (63.8)	214 (125–291)	210 (103)		239 (128–280)	205 (116)
Lactate (mmol/l)	1.30 (0.90–2.20)	1.61 (1.05)	1.20 (0.80–1.90)	1.34 (0.72)		4.10 (1.90–19)	9.73 (10.9)
pH	7.44 (7.40–7.46)	7.43 (0.04)	7.41 (7.39–7.43)	7.41 (0.04)		7.32 (7.29–7.36)	7.31 (0.05)
Hemoglobin (g/dl)	9.10 (8.40–10.2)	9.37 (1.31)	9.30 (7.70–10.8)	9.67 (2.56)		9.20 (8.60–9.60)	9.58 (1.58)
Creatinine (mg/dl)	0.96 (0.73–1.14)	1.41 (1.34)	1.22 (0.81–1.50)	1.50 (1.13)	2 (8.70%)	2.85 (1.43–3.38)	2.67 (1.19)
Total bilirubin (µmol/l)	14.9 (6.50–19.0)	18.1 (23.6)	14.0 (7.70–25.3)	17.8 (15.8)		21.7 (15.4–30.6)	30.7 (26.2)
Platelets (10^9/l)	207 (159–292)	238 (86.4)	209 (135–313)	250 (149)		135 (72–198)	189 (194)
International Normalized Ratio (INR)	1.11 (1.08–1.15)	1.14 (0.17)	1.13 (1.09–1.28)	1.30 (0.40)		1.41 (1.22–1.72)	1.69 (0.88)
**Vital signs**							
Temperature (°C)	36.4 (36–36.8)	36.3 (0.83)	36.7 (36.2–37.1)	36.6 (0.71)		36.5 (36.2–38.2)	36.9 (1.30)
Horovitz Index (mmHG)	390 (343–519)	414 (160)	376 (178–514)	354 (162)	2 (8.70%)	410 (230–453)	357 (147)
Mean arterial pressure (mmHG)	76 (70–95)	81.4 (15.5)	82 (77–84)	80.7 (9.27)		73 (67–80)	73.1 (11.8)

Table S5 **Clinical characteristics for the "multiplex immunoassay" subcohort**

	Total (norm.)	SIRS vs Sepsis	SIRS vs Septic shock	Sepsis vs Septic shock
	p	p	p	p
Age (years)	0.2309	0.9832	0.1598	0.1664
Male		0.8017	0.7036~	1.0000~
Admitting department		0.3724~	0.7324~	0.5473~
Diabetes		0.2317~	0.1155~	0.0101~
Cardiovascular disease		0.9438	1.0000~	1.0000~
Respiratory disease		0.6086~	0.5172~	1.0000~
Alcohol abuse		0.4773~	1.0000~	
Acute kidney injury		0.6221	0.0016~	0.0049~
Current tumor disease		0.0069	0.0142~	0.6401~
Hospital mortality		1.0000~	0.0002~	0.0004~
Hospital length of stay (days)	0.0003	0.7187	0.8469	0.6436
SOFA score	<.0001	0.9732	<.0001	<.0001
Mechanical ventilation		1.0000~	<.0001~	0.0001~
Vasopressor therapy		0.2076	0.0710~	0.0117~
White blood cell count (10^9/l)	<.0001	0.0429	0.0980	0.2944
CRP (mg/l)	0.0434	0.0001	0.0295	0.9027
Lactate (mmol/l)	<.0001	0.3216	0.0562	0.0498
pH	0.0256	0.0464	<.0001	0.0004
Hemoglobin (g/dl)	0.0110	0.6249	0.7296	0.9080
Creatinine (mg/dl)	<.0001	0.8128	0.0198	0.0241
Total bilirubin (µmol/l)	<.0001	0.9631	0.2347	0.1968
Platelets (10^9/l)	0.0016	0.7267	0.4893	0.4099
International Normalized Ratio (INR)	<.0001	0.1042	0.0980	0.2252
Temperature (°C)	0.4155	0.2076	0.2022	0.4791
Horovitz Index (mmHG)	0.0872	0.2281	0.3524	0.9587
Mean arterial pressure (mmHG)	0.8310	0.8705	0.1270	0.1071

p-value: t-test (method Satterthwaite) for continuous parameters, #: Mann-Whitney-Wilcoxon test (U test); Chi² test for categorical parameters, ~: Fisher's exact test

Table S6 **Clinical characteristics for the "QuantiGene^{TM} Plex" subcohort**

	Total (N=15)	
	Median (IQR) n (%)	Mean (SD)
**Demographic data**		
Age (years)	71 (61–81)	69.6 (13.5)
Male	8 (53.3%)	
**Admitting department**		
General surgery	7 (46.7%)	
Gynecology	1 (6.67%)	
Neurosurgery	3 (20.0%)	
Urology	4 (26.7%)	
**Concomitant diseases**		
Diabetes	3 (20.0%)	
Cardiovascular disease	6 (40.0%)	
Respiratory disease	0 (0%)	
Alcohol abuse	0 (0%)	
Acute kidney injury	6 (40.0%)	
Current tumor disease	7 (46.7%)	
**Hospital mortality**	7 (46.7%)	
**Hospital length of stay (days)**	13.4 (8.18–41.3)	21.3 (18.8)
**SOFA score**	5 (3–9)	6.33 (4.27)
**Mechanical ventilation**	6 (40.0%)	
**Vasopressor therapy**	11 (73.3%)	
**Lab parameters**		
White blood cell count (10^9/l)	13.6 (11.6–16.6)	20.3 (16.4)
CRP (mg/l)	209 (98–276)	182 (98.2)

Table S6 **Clinical characteristics for the "QuantiGene^{TM} Plex" subcohort**

	**Total (N=15)**	
	**Median (IQR) n (%)**	**Mean (SD)**
Lactate (mmol/l)	1.50 (0.90–3.60)	3.07 (4.63)
pH	7.42 (7.36–7.46)	7.40 (0.06)
Hemoglobin (g/dl)	9 (7.80–11.5)	9.45 (1.97)
Creatinine (mg/dl)	1.19 (0.79–3.18)	1.85 (1.27)
Total bilirubin (µmol/l)	15.2 (8.89–29.1)	28.0 (32.9)
Platelets (10^9/l)	215 (137–274)	232 (149)
International Normalized Ratio (INR)	1.16 (1.08–1.25)	1.23 (0.22)
**Vital signs**		
Temperature (°C)	36.7 (36.1–37.3)	36.8 (1.13)
Horovitz Index (mmHG)	410 (350–483)	418 (136)
Mean arterial pressure (mmHG)	77 (69–91)	80.3 (17.6)

Table S6 **Clinical characteristics for the "QuantiGene^{TM} Plex" subcohort**

	SIRS (N=5)		Sepsis (N=5)		Septic shock (N=5)	
	Median (IQR) n (%)	Mean (SD)	Median (IQR) n (%)	Mean (SD)	Median (IQR) n (%)	Mean (SD)
**Demographic data**						
Age (years)	61 (45–72)	58.6 (14.1)	71 (70–74)	71.4 (7.92)	81 (68–87)	78.8 (10.9)
Male	2 (40.0%)		2 (40.0%)		4 (80.0%)	
**Admitting department**						
General surgery	1 (20.0%)		3 (60.0%)		3 (60.0%)	
Gynecology	1 (20.0%)					
Neurosurgery	2 (40.0%)				1 (20.0%)	
Urology	1 (20.0%)		2 (40.0%)		1 (20.0%)	
**Concomitant diseases**						
Diabetes	0 (0%)		1 (20.0%)		2 (40.0%)	
Cardiovascular disease	2 (40.0%)		1 (20.0%)		3 (60.0%)	
Respiratory disease	0 (0%)		0 (0%)		0 (0%)	
Alcohol abuse	0 (0%)		0 (0%)		0 (0%)	
Acute kidney injury	0 (0%)		1 (20.0%)		5 (100%)	
Current tumor disease	3 (60.0%)		3 (60.0%)		1 (20.0%)	
**Hospital mortality**	1 (20.0%)		1 (20.0%)		5 (100%)	
**Hospital length of stay (days)**	41.3 (8.18–55.0)	33.6 (25.0)	12.8 (11.2–13.4)	12.6 (2.58)	17.3 (5.55–18.2)	17.7 (17.6)
**SOFA score**	4 (3–5)	3.80 (1.92)	3 (3–4)	3.80 (1.92)	10 (9–14)	11.4 (2.88)
**Mechanical ventilation**	1 (20.0%)		1 (20.0%)		4 (80.0%)	
**Vasopressor therapy**	4 (80.0%)		2 (40.0%)		5 (100%)	
**Lab parameters**						
White blood cell count (10^9/l)	16.0 (13.0–16)	14.8 (1.96)	12.1 (11.6–13.6)	15.4 (8.76)	16.1 (10.1–58.1)	30.6 (25.7)
CRP (mg/l)	102 (76–162)	118 (87.9)	275 (137–281)	216 (91.5)	239 (209–276)	213 (98.7)

Table S6 Clinical characteristics for the "QuantiGene^{TM} Plex" subcohort

	SIRS (N=5)		Sepsis (N=5)		Septic shock (N=5)	
	Median (IQR) n (%)	Mean (SD)	Median (IQR) n (%)	Mean (SD)	Median (IQR) n (%)	Mean (SD)
Lactate (mmol/l)	1.40 (0.90–2.20)	1.78 (1.16)	1 (0.80–1.10)	1.02 (0.44)	4.10 (1.90–5.50)	6.40 (7.23)
pH	7.46 (7.44–7.47)	7.46 (0.03)	7.42 (7.40–7.43)	7.42 (0.03)	7.33 (7.31–7.36)	7.33 (0.04)
Hemoglobin (g/dl)	8.30 (7.80–9.60)	9.12 (2.00)	7.90 (7.70–9)	9.02 (2.30)	9.20 (9.10–11.5)	10.2 (1.81)
Creatinine (mg/dl)	0.79 (0.73–0.89)	0.85 (0.21)	1.04 (0.94–2)	1.77 (1.45)	3.18 (2.85–3.38)	2.92 (0.90)
Total bilirubin (µmol/l)	16.2 (6.33–28.9)	34.6 (46.0)	12.5 (8.89–15.2)	13.9 (9.47)	28.1 (14.2–30.6)	35.5 (34.6)
Platelets (10^9/l)	206 (159–257)	209 (55.7)	256 (235–439)	354 (177)	135 (57–137)	132 (109)
International Normalized Ratio (INR)	1.08 (1.01–1.11)	1.07 (0.06)	1.16 (1.12–1.24)	1.17 (0.08)	1.41 (1.23–1.56)	1.45 (0.26)
**Vital signs**						
Temperature (°C)	36.7 (36.5–36.7)	36.6 (0.40)	36.4 (35.6–36.9)	36.2 (1.01)	38.2 (36.5–38.6)	37.5 (1.47)
Horovitz Index (mmHG)	363 (350–371)	416 (155)	457 (410–471)	406 (145)	453 (410–483)	430 (138)
Mean arterial pressure (mmHG)	86 (69–107)	85.6 (21.8)	84 (81–91)	88.6 (13.0)	70 (64–73)	66.8 (9.26)

Table S6 **Clinical characteristics for the "QuantiGene^{TM} Plex" subcohort**

	Total (norm.)	SIRS vs Sepsis	SIRS vs Septic shock	Sepsis vs Septic shock
	p	p	p	p
Age (years)	0.4497	0.1245	0.0367	0.2581
Male		1.0000~	0.5238~	0.5238~
Admitting department		0.3810~	0.7143~	1.0000~
Diabetes		1.0000~	0.4444~	1.0000~
Cardiovascular disease		1.0000~	1.0000~	0.5238~
Acute kidney injury		1.0000~	0.0079~	0.0476~
Current tumor disease		1.0000~	0.5238~	0.5238~
Hospital mortality		1.0000~	0.0476~	0.0476~
Hospital length of stay (days)	0.0056	0.1343	0.2825	0.5583
SOFA score	0.1203	1.0000	0.0018	0.0018
Mechanical ventilation		1.0000~	0.2063~	0.2063~
Vasopressor therapy		0.5238~	1.0000~	0.1667~
White blood cell count (10^9/l)	<.0001	0.8852	0.2406	0.2663
CRP (mg/l)	0.1035	0.1200	0.1450	0.9589
Lactate (mmol/l)	<.0001	0.2274	0.2278	0.1716
pH	0.6897	0.0476	0.0002	0.0027
Hemoglobin (g/dl)	0.0115	0.9434	0.3968	0.3946
Creatinine (mg/dl)	0.0101	0.2317	0.0056	0.1746
Total bilirubin (µmol/l)	0.0001	0.3765	0.9744	0.2419
Platelets (10^9/l)	0.0867	0.1431	0.2090	0.0497
International Normalized Ratio (INR)	0.0083	0.0621	0.0277	0.0684
Temperature (°C)	0.7270	0.4037	0.2486	0.1362
Horovitz Index (mmHG)	0.9877	0.9162	0.8848	0.7935
Mean arterial pressure (mmHG)	0.5066	0.7997	0.1316	0.0178

p- value: t-test (method Satterthwaite) for continuous parameters, Chi² test for categorical parameters, ~: Fisher's exact test

Table S7 **Clinical characteristics for the “qRT-PCR total granulocytes” subcohort**

	Total (N=30)		
	**Median (IQR) n (%)**	**Mean (SD)**	**Missing n (%)**
**Demographic data**			
Age (years)	70 (61–74)	67.2 (13.0)	
Male	18 (60.0%)		
**Admitting department**			
Dermatology	1 (3.33%)		
General surgery	16 (53.3%)		
Gynecology	1 (3.33%)		
Neurosurgery	2 (6.67%)		
Orthopedics and trauma center	2 (6.67%)		
Urology	8 (26.7%)		
**Concomitant diseases**			
Diabetes	3 (10.0%)		
Cardiovascular disease	15 (50.0%)		
Respiratory disease	4 (13.3%)		
Alcohol abuse	1 (3.33%)		
Acute kidney injury	10 (33.3%)		
Current tumor disease	15 (50.0%)		
**Hospital mortality**	3 (10.0%)		
**Hospital length of stay (days)**	15.7 (10.3–31.6)	22.1 (16.1)	
**SOFA score**	4 (3–5)	4.23 (2.11)	
**Mechanical ventilation**	4 (13.3%)		
**Vasopressor therapy**	17 (56.7%)		
**Lab parameters**			

Table S7 Clinical characteristics for the "qRT-PCR total granulocytes" subcohort

	Total (N=30)		
	**Median (IQR) n (%)**	**Mean (SD)**	**Missing n (%)**
White blood cell count (10^9/l)	15.6 (9.40–17.3)	15.9 (8.32)	
CRP (mg/l)	131 (79–252)	163 (104)	
Lactate (mmol/l)	1.40 (1.10–2.20)	1.62 (0.91)	
pH	7.42 (7.40–7.45)	7.42 (0.04)	
Hemoglobin (g/dl)	9.05 (8–10.4)	9.55 (1.93)	
Creatinine (mg/dl)	1.05 (0.79–1.47)	1.38 (1.05)	
Total bilirubin (µmol/l)	14.1 (7.70–20.7)	20.5 (22.6)	
Platelets (10^9/l)	239 (159–292)	255 (124)	
International Normalized Ratio (INR)	1.13 (1.09–1.23)	1.19 (0.21)	
**Vital signs**			
Temperature (°C)	36.7 (36.3–37)	36.6 (0.65)	
Horovitz Index (mmHG)	374 (294–417)	367 (130)	2 (6.67%)
Mean arterial pressure (mmHG)	83.5 (70–91)	82.3 (14.6)	

Table S7 **Clinical characteristics for the “qRT-PCR total granulocytes” subcohort**

	SIRS (N=16)			Sepsis (N=14)		
	Median (IQR) n (%)	Mean (SD)	Missing n (%)	Median (IQR) n (%)	Mean (SD)	Missing n (%)
**Demographic data**						
Age (years)	70 (58.5–72)	65.4 (11.6)		71 (64–82)	69.3 (14.7)	
Male	9 (56.3%)			9 (64.3%)		
**Admitting department**						
Dermatology	1 (6.25%)					
General surgery	7 (43.8%)			9 (64.3%)		
Gynecology	1 (6.25%)					
Neurosurgery	2 (12.5%)					
Orthopedics and trauma center	1 (6.25%)			1 (7.14%)		
Urology	4 (25.0%)			4 (28.6%)		
**Concomitant diseases**						
Diabetes	2 (12.5%)			1 (7.14%)		
Cardiovascular disease	9 (56.3%)			6 (42.9%)		
Respiratory disease	2 (12.5%)			2 (14.3%)		
Alcohol abuse	1 (6.25%)			0 (0%)		
Acute kidney injury	5 (31.3%)			5 (35.7%)		
Current tumor disease	10 (62.5%)			5 (35.7%)		
**Hospital mortality**	1 (6.25%)			2 (14.3%)		
**Hospital length of stay (days)**	20.8 (12.8–37.7)	26.1 (16.9)		12.7 (9.45–19.1)	17.6 (14.5)	
**SOFA score**	4 (2.50–5)	3.88 (1.63)		4 (3–6)	4.64 (2.56)	
**Mechanical ventilation**	1 (6.25%)			3 (21.4%)		
**Vasopressor therapy**	10 (62.5%)			7 (50.0%)		
**Lab parameters**						

Table S7 **Clinical characteristics for the "qRT-PCR total granulocytes" subcohort**

	SIRS (N=16)			Sepsis (N=14)		
	**Median (IQR) n (%)**	**Mean (SD)**	**Missing n (%)**	**Median (IQR) n (%)**	**Mean (SD)**	**Missing n (%)**
White blood cell count (10^9/l)	14.2 (10.4–16.0)	13.3 (3.52)		16.8 (9.40–26.1)	18.9 (11.0)	
CRP (mg/l)	87 (67–106)	94.6 (58.4)		256 (203–291)	240 (90.0)	
Lactate (mmol/l)	1.45 (1–2.20)	1.77 (1.11)		1.35 (1.10–1.90)	1.45 (0.61)	
pH	7.43 (7.40–7.46)	7.43 (0.03)		7.41 (7.39–7.43)	7.41 (0.04)	
Hemoglobin (g/dl)	8.90 (7.95–10.2)	9.30 (1.64)		9.35 (8–10.4)	9.84 (2.25)	
Creatinine (mg/dl)	0.99 (0.76–1.17)	1.26 (1.14)		1.26 (0.81–2)	1.51 (0.97)	
Total bilirubin (µmol/l)	14.8 (6.41–19.4)	20.1 (26.4)		14.1 (9.75–25.3)	20.9 (18.4)	
Platelets (10^9/l)	256 (170–301)	256 (89.5)		208 (135–292)	254 (158)	
International Normalized Ratio (INR)	1.11 (1.07–1.14)	1.14 (0.20)		1.21 (1.13–1.25)	1.25 (0.21)	
**Vital signs**						
Temperature (°C)	36.6 (36.1–36.8)	36.5 (0.50)		36.8 (36.4–37.1)	36.7 (0.80)	
Horovitz Index (mmHG)	363 (307–400)	365 (115)	1 (6.25%)	381 (240–514)	369 (150)	1 (7.14%)
Mean arterial pressure (mmHG)	76.5 (69.5–98)	81.7 (16.6)		84 (77–89)	83.1 (12.3)	

Table S7 **Clinical characteristics for the “qRT-PCR total granulocytes” subcohort**

	SIRS vs Sepsis
	**p**
Age (years)	0.4375
Male	0.6540
Admitting department	0.7490~
Diabetes	1.0000~
Cardiovascular disease	0.4642
Respiratory disease	1.0000~
Alcohol abuse	1.0000~
Acute kidney injury	1.0000~
Current tumor disease	0.1432
Hospital mortality	0.5862~
Hospital length of stay (days)	0.1500
SOFA score	0.3455
Mechanical ventilation	0.3155~
Vasopressor therapy	0.4906
White blood cell count (10^9/l)	0.0865
CRP (mg/l)	<.0001
Lactate (mmol/l)	0.3239
pH	0.0737
Hemoglobin (g/dl)	0.4687
Creatinine (mg/dl)	0.5355
Total bilirubin (µmol/l)	0.9235
Platelets (10^9/l)	0.9698
International Normalized Ratio (INR)	0.1775
Temperature (°C)	0.4035
Horovitz Index (mmHG)	0.9333
Mean arterial pressure (mmHG)	0.7963

p- value: t-test (method Satterthwaite) for continuous parameters, Chi² test for categorical parameters, ~: Fisher's exact test

Table S8 **Clinical characteristics for the "qRT-PCR HD & LD granulocytes" subcohort**

	**Total (N=25)**		
	**Median (IQR) n (%)**	**Mean (SD)**	**Missing n (%)**
**Demographic data**			
Age (years)	70 (62–74)	68.6 (11.8)	
Male	14 (56.0%)		
**Admitting department**			
Dermatology	1 (4.00%)		
General surgery	12 (48.0%)		
Gynecology	1 (4.00%)		
Neurosurgery	2 (8.00%)		
Orthopedics and trauma center	2 (8.00%)		
Urology	7 (28.0%)		
**Concomitant diseases**			
Diabetes	2 (8.00%)		
Cardiovascular disease	14 (56.0%)		
Respiratory disease	2 (8.00%)		
Alcohol abuse	0 (0%)		
Acute kidney injury	8 (32.0%)		
Current tumor disease	14 (56.0%)		
**Hospital mortality**	3 (12.0%)		
**Hospital length of stay (days)**	16.1 (10.3–34.1)	23.5 (17.1)	
**SOFA score**	4 (3–5)	4.52 (2.18)	
**Mechanical ventilation**	3 (12.0%)		
**Vasopressor therapy**	15 (60.0%)		
**Lab parameters**			

Table S8 **Clinical characteristics for the "qRT-PCR HD & LD granulocytes" subcohort**

	**Total (N=25)**		
	**Median (IQR) n (%)**	**Mean (SD)**	**Missing n (%)**
White blood cell count (10^9/l)	15.9 (11.6–17.5)	16.6 (8.74)	
CRP (mg/l)	110 (79–232)	153 (106)	
Lactate (mmol/l)	1.40 (0.90–1.90)	1.60 (0.96)	
pH	7.43 (7.40–7.46)	7.43 (0.03)	
Hemoglobin (g/dl)	8.90 (7.90–9.60)	9.29 (1.85)	
Creatinine (mg/dl)	1.04 (0.76–1.50)	1.45 (1.14)	
Total bilirubin (µmol/l)	14.2 (9.41–20.7)	21.9 (24.4)	
Platelets (10^9/l)	235 (159–274)	255 (125)	
International Normalized Ratio (INR)	1.15 (1.10–1.23)	1.21 (0.22)	
**Vital signs**			
Temperature (°C)	36.6 (36.1–36.8)	36.5 (0.65)	
Horovitz Index (mmHG)	386 (335–440)	388 (126)	1 (4.00%)
Mean arterial pressure (mmHG)	83 (70–89)	81.8 (15.2)	

Table S8 **Clinical characteristics for the "qRT-PCR HD & LD granulocytes" subcohort**

	SIRS (N=14)			Sepsis (N=11)	
	Median (IQR) n (%)	Mean (SD)	Missing n (%)	Median (IQR) n (%)	Mean (SD)
**Demographic data**					
Age (years)	70 (61–72)	66.1 (11.5)		72 (64–82)	71.7 (11.8)
Male	7 (50.0%)			7 (63.6%)	
**Admitting department**					
Dermatology	1 (7.14%)				
General surgery	5 (35.7%)			7 (63.6%)	
Gynecology	1 (7.14%)				
Neurosurgery	2 (14.3%)				
Orthopedics and trauma center	1 (7.14%)			1 (9.09%)	
Urology	4 (28.6%)			3 (27.3%)	
**Concomitant diseases**					
Diabetes	1 (7.14%)			1 (9.09%)	
Cardiovascular disease	9 (64.3%)			5 (45.5%)	
Respiratory disease	1 (7.14%)			1 (9.09%)	
Alcohol abuse	0 (0%)			0 (0%)	
Acute kidney injury	4 (28.6%)			4 (36.4%)	
Current tumor disease	9 (64.3%)			5 (45.5%)	
**Hospital mortality**	1 (7.14%)			2 (18.2%)	
**Hospital length of stay (days)**	20.8 (14.4–41.3)	27.1 (17.6)		12.8 (9.45–19.4)	19.0 (16.0)
**SOFA score**	4 (3–5)	4.07 (1.64)		4 (3–7)	5.09 (2.70)
**Mechanical ventilation**	1 (7.14%)			2 (18.2%)	
**Vasopressor therapy**	9 (64.3%)			6 (54.5%)	
**Lab parameters**					

Table S8 **Clinical characteristics for the "qRT-PCR HD & LD granulocytes" subcohort**

	SIRS (N=14)			Sepsis (N=11)	
	Median (IQR) n (%)	Mean (SD)	Missing n (%)	Median (IQR) n (%)	Mean (SD)
White blood cell count (10^9/l)	12.7 (9.10–16)	12.9 (3.65)		17.8 (11.6–30.8)	21.2 (11.1)
CRP (mg/l)	87 (59–102)	90.2 (58.1)		232 (137–291)	233 (101)
Lactate (mmol/l)	1.45 (0.90–2.20)	1.74 (1.15)		1.40 (0.80–1.90)	1.42 (0.65)
pH	7.44 (7.41–7.47)	7.44 (0.03)		7.42 (7.39–7.45)	7.42 (0.03)
Hemoglobin (g/dl)	8.75 (7.80–9.60)	9.22 (1.71)		9 (7.90–10.4)	9.38 (2.10)
Creatinine (mg/dl)	0.95 (0.73–1.19)	1.29 (1.22)		1.47 (0.76–2.08)	1.65 (1.05)
Total bilirubin (µmol/l)	14.8 (6.50–19.8)	21.3 (28.0)		14.2 (11.6–29.1)	22.7 (20.1)
Platelets (10^9/l)	249 (159–274)	241 (81.2)		209 (135–439)	273 (167)
International Normalized Ratio (INR)	1.11 (1.08–1.15)	1.15 (0.21)		1.23 (1.16–1.28)	1.28 (0.23)
**Vital signs**					
Temperature (°C)	36.6 (36.1–36.8)	36.5 (0.52)		36.6 (36.3–36.9)	36.5 (0.81)
Horovitz Index (mmHG)	371 (350–400)	380 (114)	1 (7.14%)	410 (319–518)	399 (144)
Mean arterial pressure (mmHG)	76.5 (70–101)	81.9 (16.9)		84 (69–89)	81.8 (13.4)

Table S8 **Clinical characteristics for the "qRT-PCR HD & LD granulocytes" subcohort**

	SIRS vs Sepsis
	p
Age (years)	0.2427
Male	0.6887~
Admitting department	0.6980~
Diabetes	1.0000~
Cardiovascular disease	0.4347~
Respiratory disease	1.0000~
Acute kidney injury	1.0000~
Current tumor disease	0.4347~
Hospital mortality	0.5648~
Hospital length of stay (days)	0.2390
SOFA score	0.2868
Mechanical ventilation	0.5648~
Vasopressor therapy	0.6968~
White blood cell count (10^9/l)	0.0358
CRP (mg/l)	0.0008
Lactate (mmol/l)	0.3940
pH	0.1746
Hemoglobin (g/dl)	0.8393
Creatinine (mg/dl)	0.4447
Total bilirubin (µmol/l)	0.8855
Platelets (10^9/l)	0.5673
International Normalized Ratio (INR)	0.1628
Temperature (°C)	0.8112
Horovitz Index (mmHG)	0.7250
Mean arterial pressure (mmHG)	0.9949

p- value: t-test (method Satterthwaite) for continuous parameters, Chi² test for categorical parameters, ~: Fisher's exact test

Fig. S1 Analytical patient subcohorts

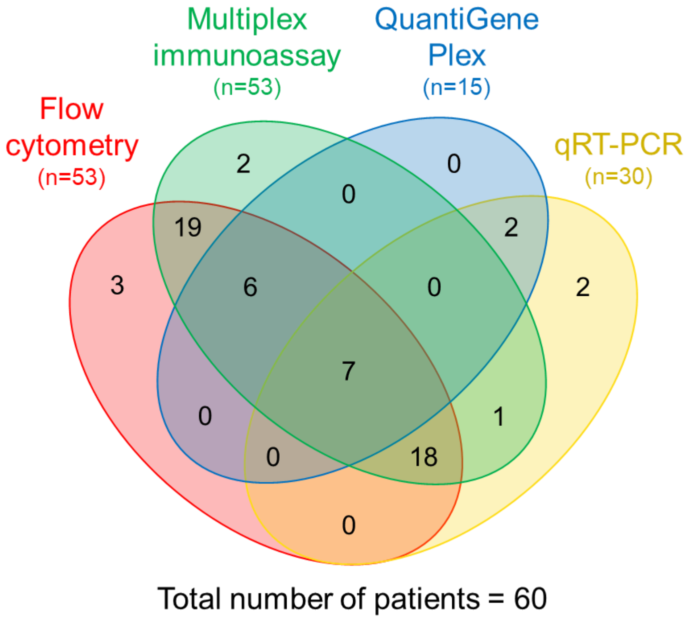

The patients contributing to flow cytometric precursor counting and precursor profiling are combined. Most blood samples contributed to both the flow cytometry and multiplex immunoassay analyses. A relatively small number of samples (15) was chosen for the Quantigene™ Plex analysis to facilitate analyzing a large test panel of 135 genes. The availability of samples for the qRT-PCR (30) was limited by the yields of the RNA preparations. The diagram was generated with InteractiVenn (Heberle H, Meirelles GV, da Silva FR, Telles GP, Minghim R: InteractiVenn: a web-based tool for the analysis of sets through Venn diagrams. BMC Bioinformatics 2015, 16(1):169). The data shown is available from heiDATA (https://doi.org/10.11588/data/JRWPFV).

Fig. S2 Flow cytometric gating strategy to identify granulocyte precursors

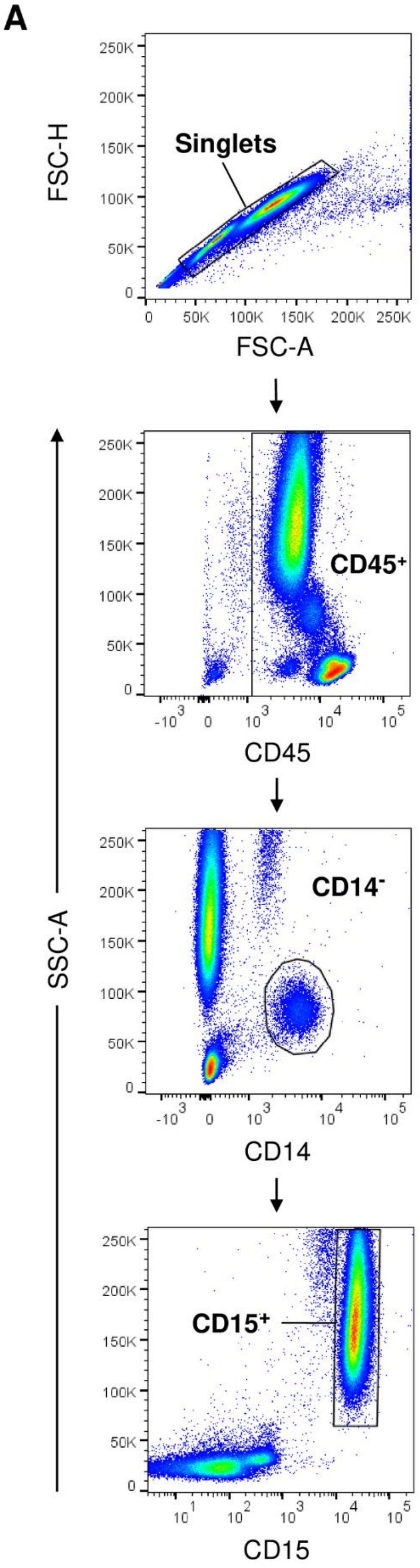

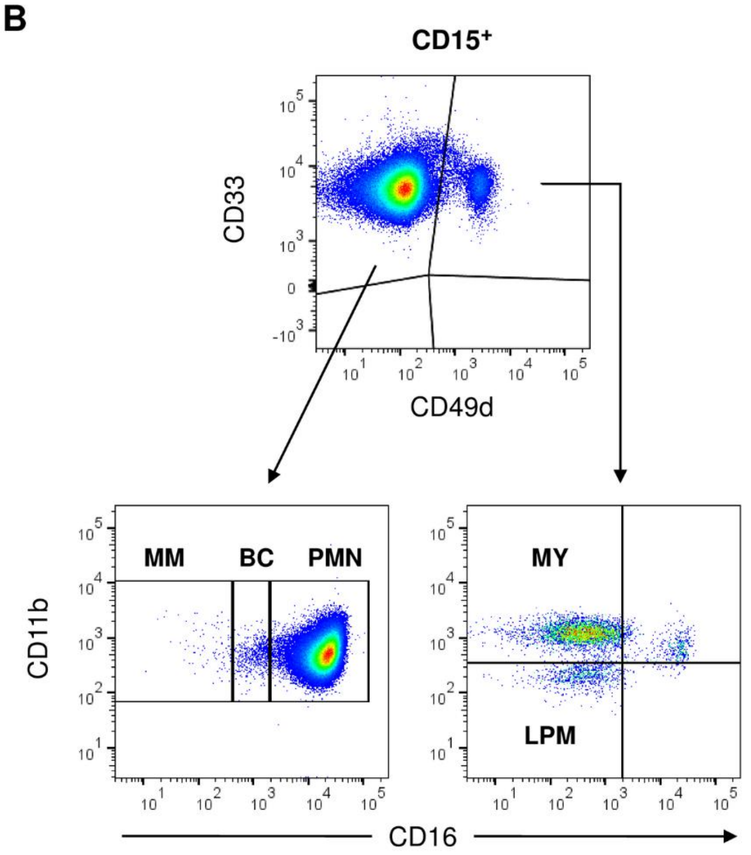

Representative dot plots illustrating the flow cytometric gating strategy for identifying stages of terminal granulocytic differentiation in whole blood (cf. Figure 5 of the main text). (**A**) Granulocytes were identified in lyzed whole blood by sequential gating on singles in a forward scatter height (FSC-H) vs. area (FSC-A) plot followed by selection of $CD45^{+}$ leukocytes, exclusion of $CD14^{+}$ monocytes and selection of $CD15^{+}$ granulocytes, excluding highly granular eosinophils, in sideward scatter area (SSC-A) plots. (**B**) $CD15^{+}$ granulocytic precursors were defined according to Pedersen et al. (J Immunol 2016; 197: 1989-1999).

**LPM**	(late promyelocyte):	$CD33^{+}CD49d^{+}CD11b^{-}CD16^{-}$
**MY**	(myelocytes):	$CD33^{+}CD49d^{+}CD11b^{+}CD16^{-}$
**MM**	(metamyelocytes):	$CD33^{+}CD49d^{-}CD11b^{+}CD16^{-}$
**BC**	(band cells):	$CD33^{+}CD49d^{-}CD11b^{+}CD16^{dim}$
**PMN**	(polymorphonuclear neutrophil):	$CD33^{+}CD49d^{-}CD11b^{+}CD16^{high}$

Fig. S3 Radar charts comparing the analytical subcohorts to the total cohort

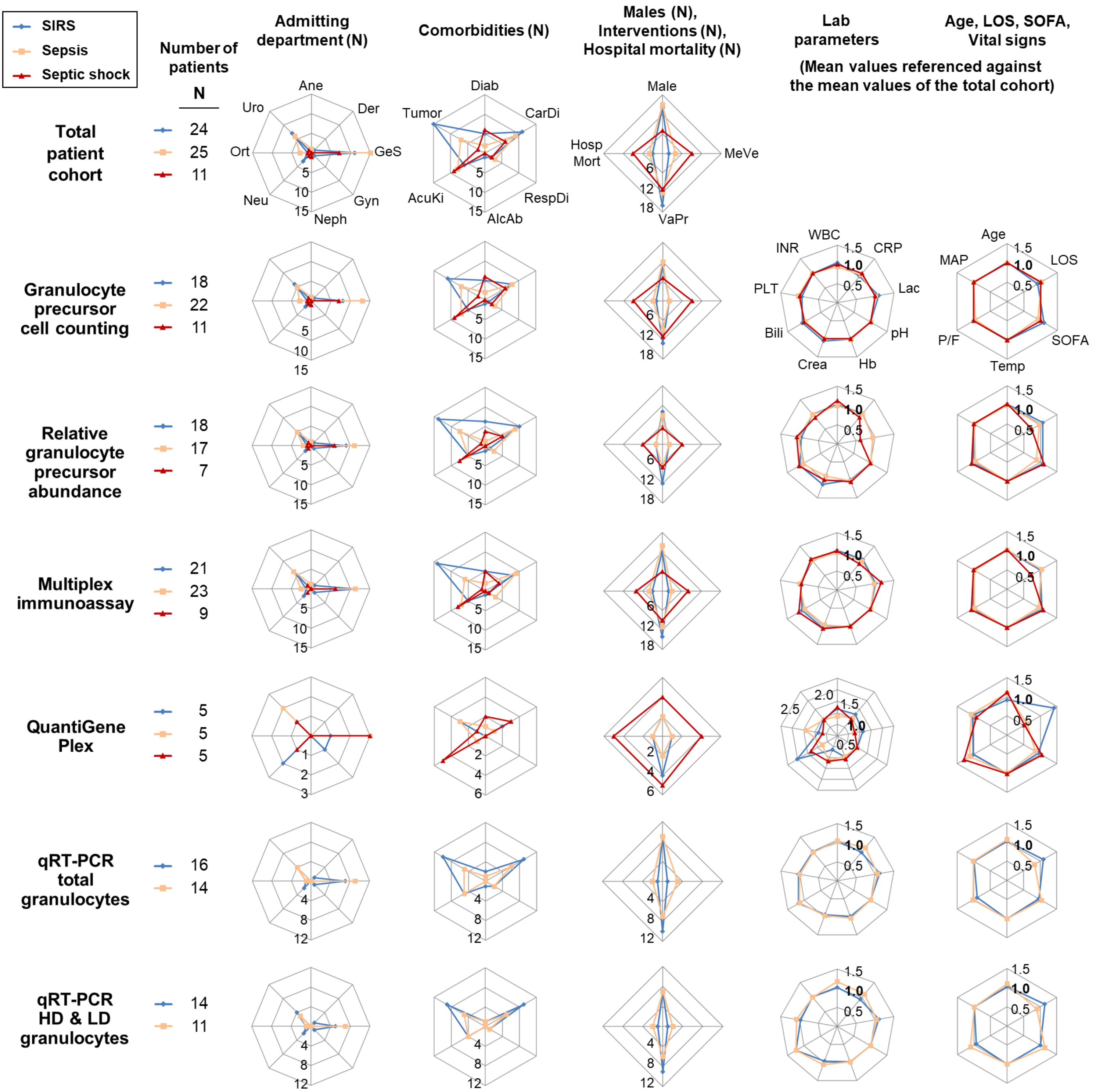

Fig. S4 Spearman's rank-order correlation for SOFA score values and granulocyte precursor blood counts

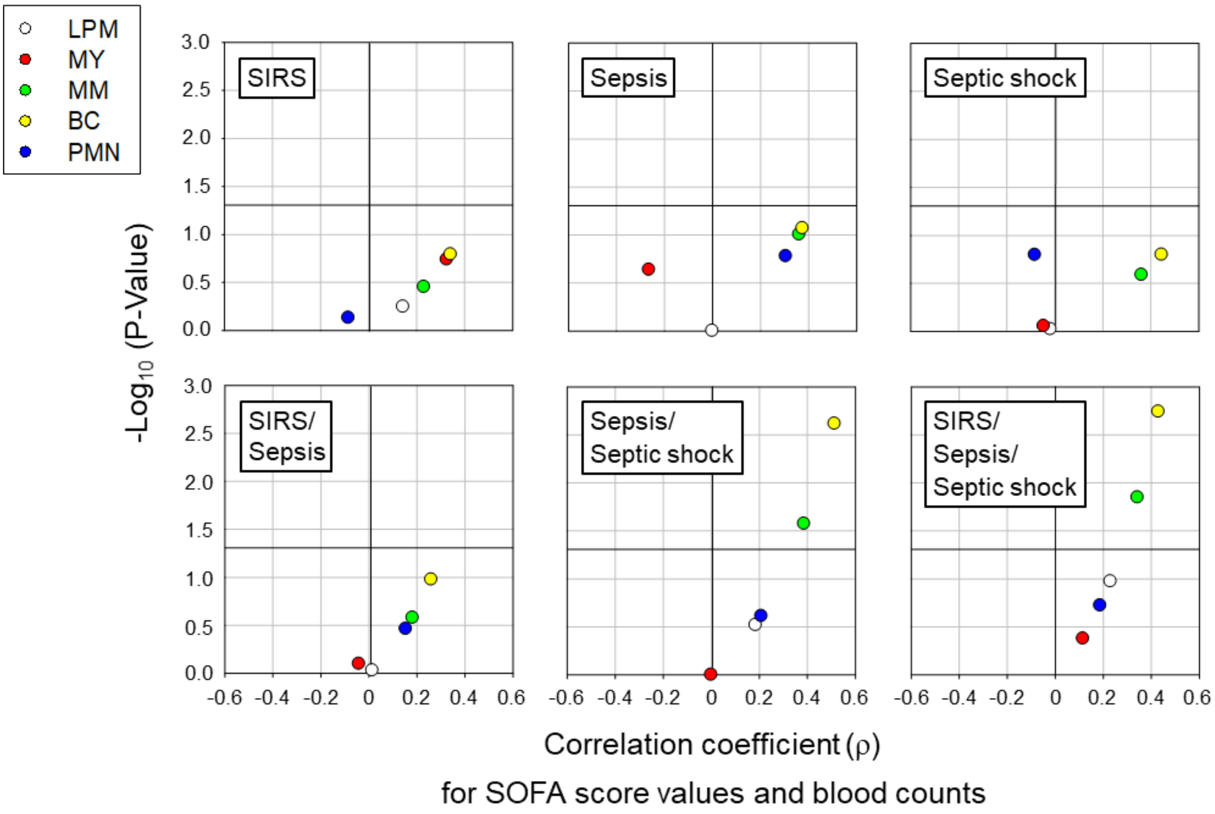

Strength and direction of correlations are measured by the Spearman's rank correlation coefficient ρ, which is plotted against the negative common logarithm of the p-value. The solid horizontal line corresponds to $p = 0.05$, the threshold for statistical significance. *SIRS*, n=18; *sepsis*, n=22; *septic shock,* n=11; LPM: late promyelocyte; MY: myelocyte; MM: metamyelocyte; BC: band cell; PMN: polymorphonuclear neutrophil.

Fig. S5 Venn diagrams for genes with differential expression between patient subgroups across total, HD and LD granulocytes

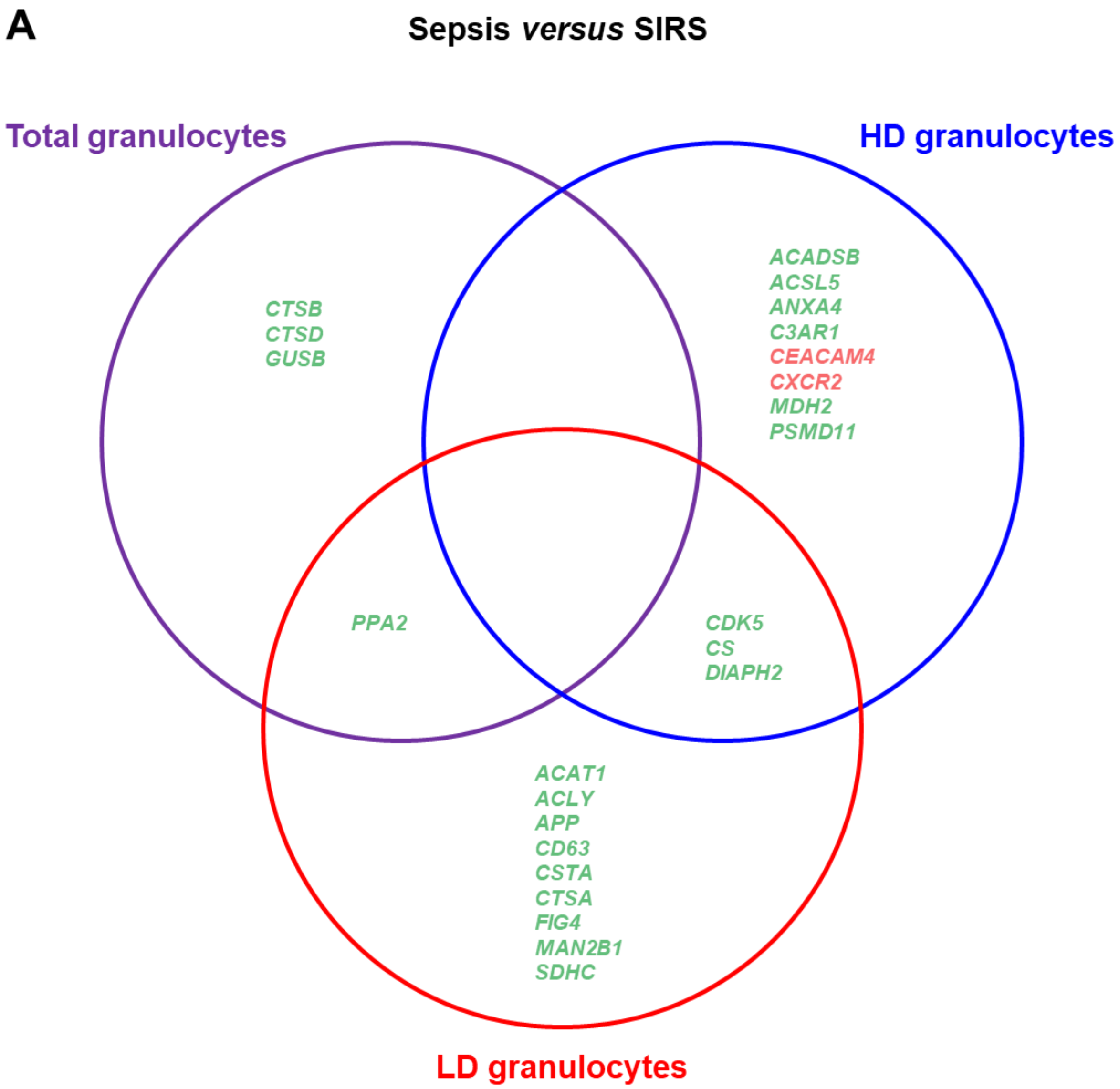

Fig. S5 continued

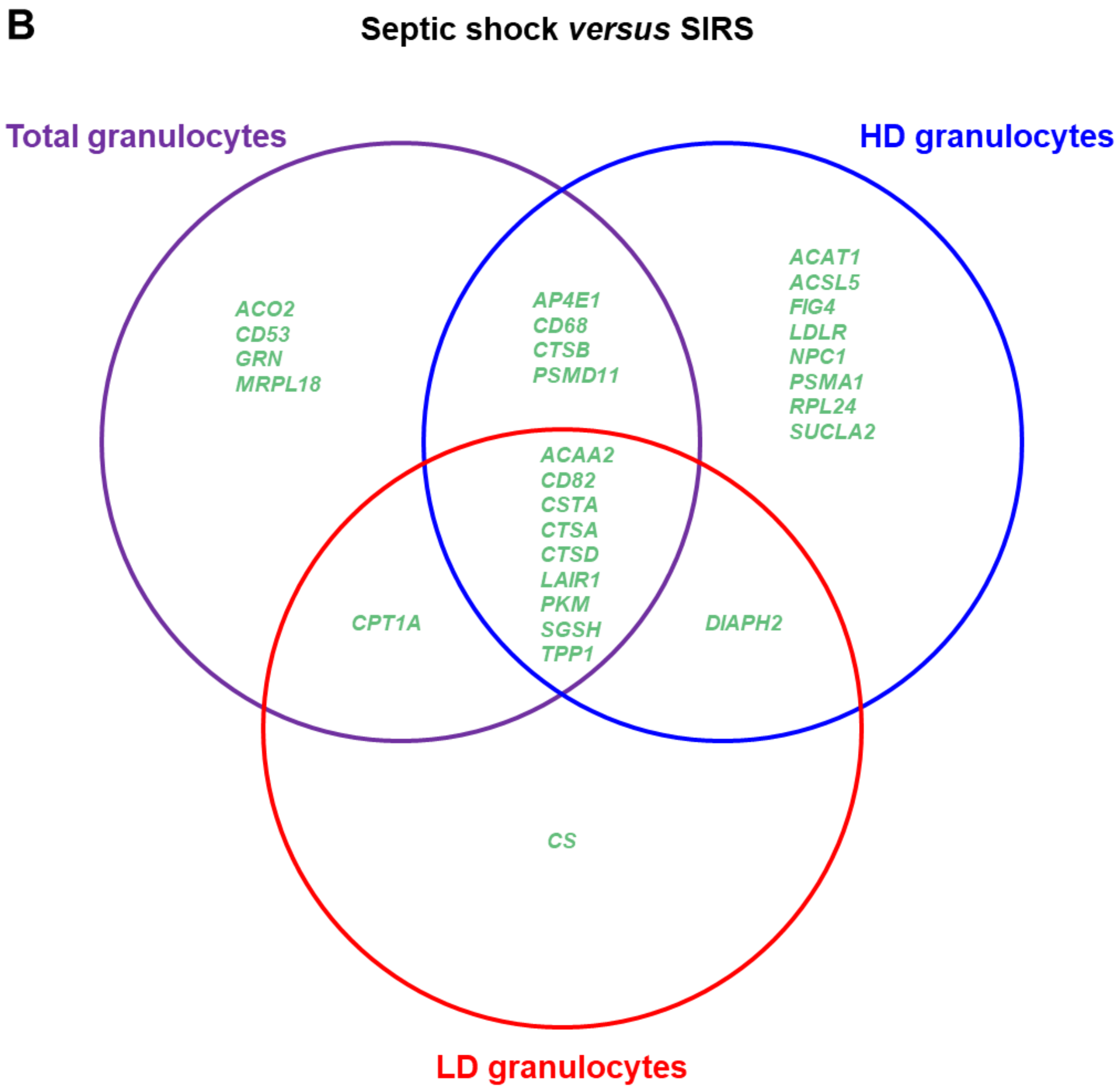

Fig. S5 continued

C

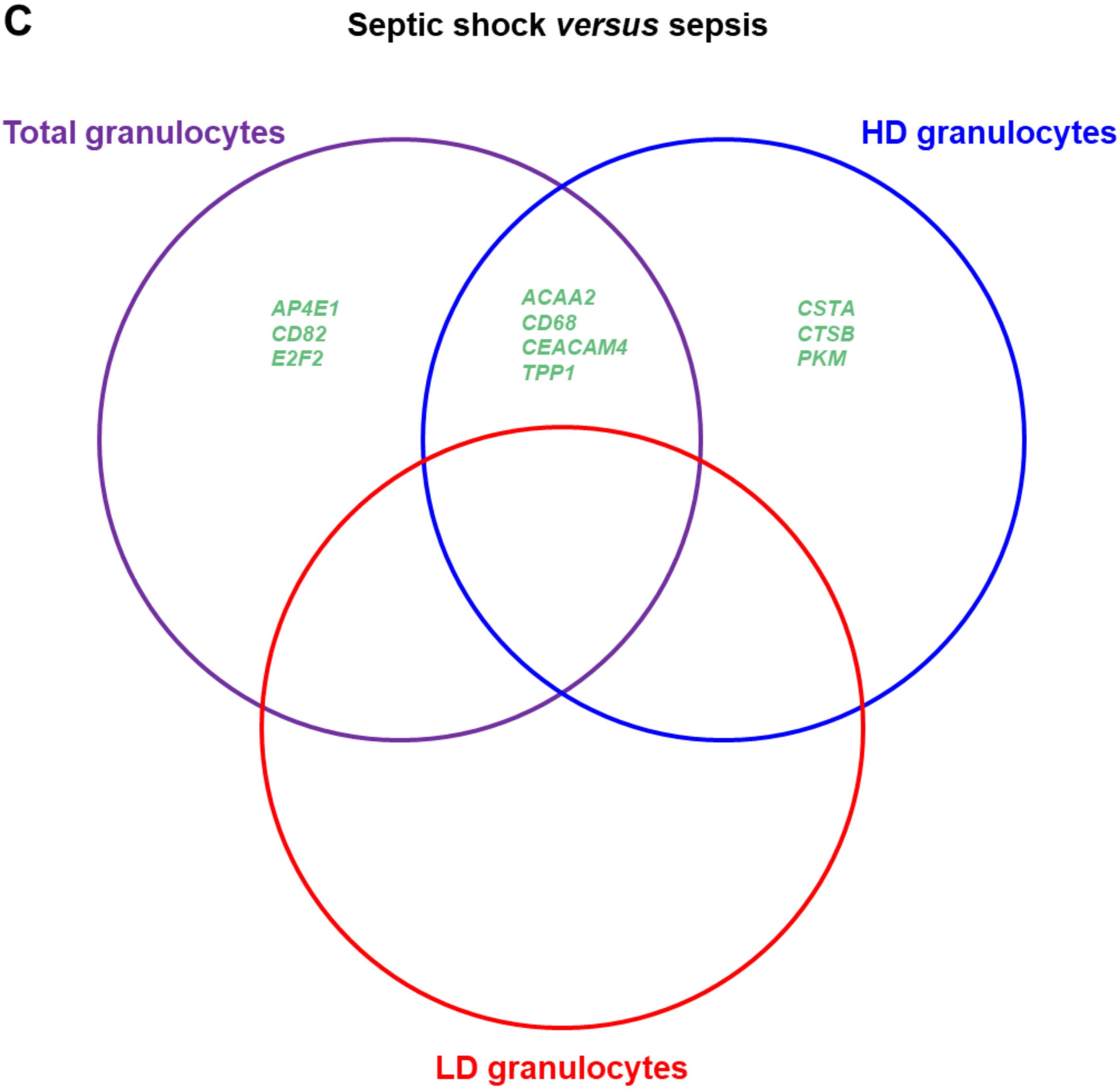

Supplement: Supplementary file 1 — Supplementary Material 1 [file 12950_2024_414_MOESM1_ESM.pdf]
